# Glareosin: a novel sexually dimorphic urinary lipocalin in the bank vole, *Myodes glareolus*

**DOI:** 10.1098/rsob.170135

**Published:** 2017-09-06

**Authors:** Grace M. Loxley, Jennifer Unsworth, Michael J. Turton, Alexandra Jebb, Kathryn S. Lilley, Deborah M. Simpson, Daniel J. Rigden, Jane L. Hurst, Robert J. Beynon

**Affiliations:** 1 Centre for Proteome Research, Institute of Integrative Biology, University of Liverpool, Liverpool L69 7ZB, UK; 2 Institute of Integrative Biology, University of Liverpool, Liverpool L69 7ZB, UK; 3 Mammalian Behaviour and Evolution Group, Institute of Integrative Biology, University of Liverpool, Leahurst Campus, Neston CH64 7TE, UK; 4 Department of Biochemistry, University of Leicester, Leicester LE1 7RH, UK

**Keywords:** bank vole, *Myodes glareoulus*, odorant-binding protein, metabolic labelling, mass spectrometry, glareosin

## Abstract

The urine of bank voles (*Myodes glareolus*) contains substantial quantities of a small protein that is expressed at much higher levels in males than females, and at higher levels in males in the breeding season. This protein was purified and completely sequenced at the protein level by mass spectrometry. Leucine/isoleucine ambiguity was completely resolved by metabolic labelling, monitoring the incorporation of dietary deuterated leucine into specific sites in the protein. The predicted mass of the sequenced protein was exactly consonant with the mass of the protein measured in bank vole urine samples, correcting for the formation of two disulfide bonds. The sequence of the protein revealed that it was a lipocalin related to aphrodisin and other odorant-binding proteins (OBPs), but differed from all OBPs previously described. The pattern of secretion in urine used for scent marking by male bank voles, and the similarity to other lipocalins used as chemical signals in rodents, suggest that this protein plays a role in male sexual and/or competitive communication. We propose the name glareosin for this novel protein to reflect the origin of the protein and to emphasize the distinction from known OBPs.

## 1. Background

Olfactory communication is prevalent in rodents, where semiochemicals are capable of transmitting information regarding identity, relatedness, territory, health status and mating availability [1–5]. Chemosignalling is highly conserved, with many species displaying scent marking behaviours that make use of urine, faeces and glandular secretions to convey information. Members of the lipocalin protein family are often involved in chemosignalling, and are found in several rodent secretions and tissues where they serve this role, including nasal tissue, saliva, urine, tears and vaginal discharge [6–9]. Murine rodents (Old World rats and mice, sub-family Murinae) express a set of proteins known as major urinary proteins (MUPs), which can be highly polymorphic, whereas hamsters and voles (sub-families Cricetinae and Arvicolinae) seem to express chemosignalling lipocalins more typical of the odorant-binding protein (OBP) family.

Urinary protein expression has been well characterized in the house mouse (*Mus musculus domesticus*). The highly polymorphic MUPs, expressed by both males and females, can communicate individual identity, kinship, dominance, and potentially oestrus and health status [10–19]. Sexual dimorphism is pronounced, with males typically expressing three- to fourfold more MUPs overall than females, while some MUPs are expressed almost exclusively by males [6]. MUPs mediate chemosignalling, either by direct detection through vomeronasal 2 (V2R) receptors in the vomeronasal organ [11] or by binding volatile components, promoting their slow release over a prolonged period, and extend the lifespan of the scent mark [13,20,21]. The protein complement of rat urine also has a similarly polymorphic expression of homologous MUPs, but with much stronger sexual dimorphism [22,23].

Relatively little is yet known about the expression of chemosignalling proteins in the vole family, but sexual dimorphism in urinary protein expression has been observed in the bank vole, *Myodes glareolus*, where protein levels are much higher in males [24]. Bank voles live in small, mixed-sex groups during the winter that break up in the breeding season. While breeding females inhabit non-overlapping home ranges close to the over-wintering site, males have larger overlapping home ranges within hierarchical groups that overlap several females [25,26]. Males deposit urine around their territories in numerous small scent marks, using long brush-like hairs on the prepuce tip to streak out their scent [27,28], contrasting with the excretion of urine in pools by females [28,29]. Scent marking rates are particularly high in new environments, while dominant males also mark subordinate male burrow and nest areas continually. Females prefer males that scent mark more frequently [30]. Three male-specific OBPs have been identified in male bank vole urine that might play a role in chemical signalling [31]. To understand the expression and potential role of urinary proteins in bank vole communication further, we examined the expression of urinary proteins in wild-caught and captive-bred voles in the breeding and non-breeding season. Here, we characterize a new urinary protein in *M. glareolus*, distinct from those previously identified, that is expressed at high level only by males and only in the breeding season. The complete protein sequence was obtained primarily using in-solution protease digestion followed by tandem mass spectrometry, distinguishing between the otherwise isobaric amino acids leucine and isoleucine using metabolic labelling. Homology modelling and structural analysis reveal strong similarity to known OBPs, but this protein is distinct from those previously described in bank voles or in other species and is the most abundant urinary protein expressed by male bank voles. Given the potentially important investment by male bank voles in this particular urinary protein during the breeding season, we propose the name glareosin to distinguish this from other OBPs.

## 2. Material and methods

### 2.1. Sampling

Urine samples were collected from both wild-caught and captive-bred *M. glareolus* voles derived from two different geographical areas of the UK (Wirral Peninsula in Merseyside, approx. 53.288° N, −3.028° E, and Kielder Forest in Northumberland, approx. 55.208° N, −2.528° E). Urine was freely expressed and collected from clean plastic cages prior to measurement of protein and creatinine concentration.

### 2.2. In-gel proteolysis

Protein bands from SDS–PAGE were digested with trypsin to generate peptides suitable for further analysis by MALDI-ToF mass spectrometry. Excised gel plugs (approx. 1 mm^3^) were destained, then reduced and carbamidomethylated. Peptides were recovered for mass spectrometric analysis.

### 2.3. Edman degradation

SDS–PAGE gels before staining were electroblotted to polyvinylidene difluoride (PVDF) membranes for N-terminal sequencing using an Applied Biosystems 476A gas-phase sequencer (Applied Biosystems). After electroblotting, the PVDF was stained with Coomassie blue to visualize protein bands prior to excision and Edman degradation.

### 2.4. MALDI-ToF mass spectrometry

Analysis of peptides from in-gel digests was undertaken using a MALDI-ToF reflectron mass spectrometer (Waters, Manchester, UK) in positive ion mode. All aspects of data acquisition, processing and machine management were controlled through the MassLynx software suite (v. 4.0).

### 2.5. In-solution proteolysis

Aliquots of protein (10 µg) purified from cage deposits by anion exchange chromatography were reduced, alkylated and digested with trypsin, endopeptidase GluC or endopeptidase LysC.

### 2.6. Electrospray ionization mass spectrometry

Electrospray ionization mass spectrometry (ESI-MS) was used in two modes: liquid chromatography–mass spectrometry (LC–MS) was used for intact mass analysis while tandem mass spectrometry (MS/MS) was used for peptide sequence analysis. All ESI-MS was undertaken on a Q-ToF Micro mass spectrometer (Waters, Manchester, UK) in positive ion mode. As an additional aid in the interpretation of tandem mass spectra, peptides were isotopically labelled with ^18^O by performing proteolytic digestion in a 1 : 1 mixture of light (H_2_[^16^O]) and heavy (H_2_[^18^O]) water. Incorporation of a 1 : 1 mixture of [^16^O] and [^18^O] atoms into the newly formed C-termini of peptides prior to tandem mass spectrometry allowed **y**-ions to be identified as a sequence of doublets of approximately equal intensity, separated by 2 Da. To confirm and complete the sequence, we repeated the digestions and analysed the samples on a high-resolution instrument with high mass accuracy and resolution for precursor and product ions. For this stage, samples were analysed using a Ultimate 3000 nano system (Dionex/Thermo Fisher Scientific, Hemel Hempstead, UK) coupled with a QExactive mass spectrometer (Thermo Fisher Scientific).

### 2.7. Use of labelled dietary leucine to discriminate isoleucine from leucine

To discriminate between isobaric leucine and isoleucine residues, we fed bank voles a diet containing stable isotope-labelled leucine. Cage-deposited urine samples were collected from four voles (day 0) before they were transferred to a new cage with the [^2^H_3_] leucine diet provided *ad libitum*. Urinary proteins were reduced, alkylated and digested with trypsin in solution, followed by LC–MS/MS analysis on the QExactive-HF (Thermo Scientific) as described above. Leucine and isoleucine residues were then manually assigned from the raw data and confirmed with MASCOT and PEAKS searches under the same search conditions as below with triple labelling with deuterium as an additional variable modification, against the derived sequence of glareosin.

### 2.8. Protein sequence analysis

The final amino acid sequence was used in a BLAST search [33] using default parameters for protein matches against Rodenta. The 138 matches were reduced and processed as follows. First, incomplete sequences, sequences substantially larger than the core lipocalin size of approx. 160 amino acids or those that only matched across part of the sequence were eliminated. Some sequence entries were exact duplicates and were reduced to single entries. Finally, because we wished to compare the glareosin-secreted protein sequence, signal peptides were removed, either guided by the feature entry in the database entry or through the SignalP 4.1 server [34] (http://www.cbs.dtu.dk/services/SignalP/). The reduced sequence set was aligned with mafft using the high accuracy linsi algorithm [35] with Jalview [36] used to display and manipulate sequence alignments.

### 2.9. Phylogenetic analysis

The evolutionary history was inferred by using the maximum-likelihood method based on the JTT matrix-based model [37]. Bootstrapping analysis [38] using 500 replicates was carried out. Branches corresponding to partitions reproduced in less than 50% bootstrap replicates were collapsed. All positions containing gaps and missing data were eliminated leaving a total of 112 positions in the final dataset. Evolutionary analyses were conducted in MEGA7 [39].

### 2.10. Homology modelling

The structure of mature glareosin, without its signal peptide, was modelled using the RosettaCM protocol [40]. Ten models were produced for each combination of templates and alignments. Templates were identified from a non-redundant library of PDB structures using the HHpred server [41], and modelling was done with one, five or 10 templates assessing the results quantitatively with Rosetta's own energy function and with the Prosa II [42], DOPE [43] and QMEAN [44] protein structure quality metrics. Stereochemistry was assessed with PROCHECK [45]. Structures were superimposed using GESAMT [46]. Cavities were detected and measured using the GHECOM [47] and Profunc [48] servers. PyMOL (https://www.pymol.org/) was used to visualize and manipulate structures and to produce structure figures.

## 3. Results and discussion

**Figure 1.**
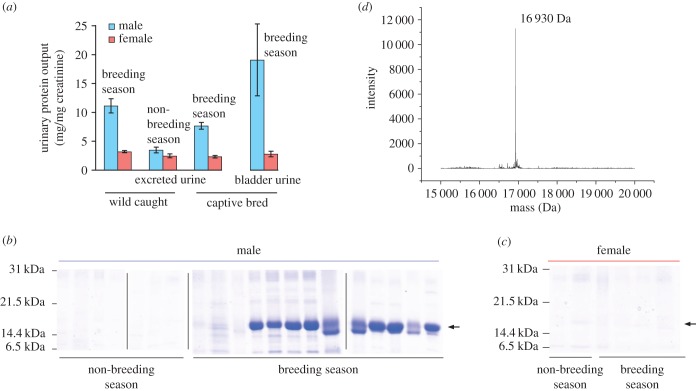
Analysis of bank vole urinary protein output. (*a*) For urine samples from adult male or female bank voles (see text), protein and creatinine concentrations were determined and expressed as mg protein (mg creatinine)^−1^ to correct for urine dilution. The protein complement was analysed by SDS–PAGE for (*b*) male and (*c*) female voles (vertical bars separate discrete gels) and by (*d*) electrospray ionization mass spectrometry (male).

To assess seasonal and sex variation in urinary protein output, urine samples were obtained from wild-caught bank voles captured during the breeding and non-breeding season (to correct for differences in urine dilution, protein output was expressed as mg (mg creatinine)^−1^). These analyses confirmed that urinary protein output was substantially higher in males, but only during the breeding season (interaction between season and sex, *F*_1,21_ = 5.19, *p* = 0.033; figure 1*a*). Male average protein output increased over threefold, from 3.5 ± 0.5 mg protein (mg creatinine)^−1^ during the non-breeding season (uncorrected urinary protein concentration 0.36 ± 0.07 mg ml^−1^) up to 11.2 ± 1.2 mg protein (mg creatinine)^−1^ in the breeding season (uncorrected urinary protein concentration 1.76 ± 0.27 mg ml^−1^). As urinary creatinine levels were not influenced by season or sex, these differences in urinary protein output were entirely due to differences in the concentration of protein excreted in urine. A preliminary assessment of protein complexity in these samples by one-dimensional (1D) SDS–PAGE revealed an intense band between 14 and 21 kDa that was evident only in male samples and only during the breeding season (figure 1*b*,*c*). We also assessed protein output in urine samples from bank voles bred in captivity and kept under breeding season lighting conditions but without sexual experience. This confirmed a highly significant sex difference in urine protein output (*F*_1,28_ = 79.6, *p* < 0.0001), with levels comparable to those seen in wild-caught voles during the breeding season (figure 1*a*). Thus, elevated protein output in males was not dependent on sexual experience. This elevated protein output was evident in male bladder urine, sampled when older voles were culled (effect of sex, *F*_1,10_ = 6.8, *p* = 0.026). SDS–PAGE confirmed that the same intense band between 14 and 21 kDa was present in male but not in female samples, in both naturally deposited and bladder urine (data not shown).

Intact mass analysis has also been used to assess the heterogeneity of urinary proteins in both captive-bred [49–52] and wild-caught mice [10,17,53,54], identifying small mass changes caused by discrete amino acid substitutions in the protein sequence. The intact mass profile of the *M. glareolus* urinary proteins was analysed by ESI-MS (figure 1*d*). A single predominant intact mass was measured in all samples at 16 930 ± 1 Da, and there was no evidence of inter- or intra-individual heterogeneity in the mass profile. The protein identified at a mass of 16 930 Da in all *M. glareolus* urine samples was purified by anion exchange chromatography. This ion exchange purified protein was recovered and used for primary sequence analysis, as genomic or transcriptomic data were lacking. The measured intact mass differed from the predicted masses of the three urinary OBP proteins reported in bank voles by Stopková *et al.* [31], which, allowing for the formation of two putative disulfide bonds, together with loss of signal peptide predicted by signalP [34], were OBP1 (D3VW62_MYOGA): 16 643 Da, OBP2 (D3VW64_MYOGA): 16 837 Da and OBP3 (D3VW62_MYOGA): 16 749 Da, consistent with this being a novel protein.

After 1D SDS–PAGE and blotting to PVDF membrane, the 16 930 Da protein was partially sequenced by gas-phase Edman degradation. Although less commonly used today, Edman degradation permits precise positioning of the true N-terminal sequence of the protein. The recovered sequence HSEIDEKWVTVAIAADNVNK used in searching (BlastP) [55] with standard search parameters aligned most strongly to the N-terminal sequence of a prairie vole (*Microtus ochrogaster*) aphrodisin-like protein 1 (best match: XP_005372052; 70% identity) and a bank vole (*M. glareolus*) OBP1 (best match, D3VW62_MYOGA; 65% identity) as well as other members of the lipocalin family. This match pointed to the potential role of this urinary protein as a semiochemical lipocalin. Although the N-terminal sequence overlapped with the first structurally conserved GlyXxxTrp region of the lipocalin family (GXW [56]), the highly conserved glycine residue of the motif was absent. However, Glu (E) and Gly (G) elute in close proximity in Edman degradation, raising the possibility of a mis-call at this position.

**Figure 2.**
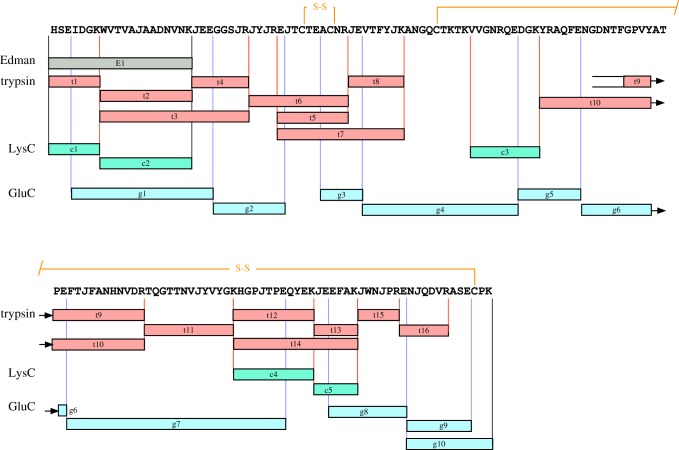
Complete amino sequence of the novel bank vole urinary protein. The bank vole urinary protein was digested with multiple endopeptidases (t, trypsin; c, endopeptidase LysC; g, endopeptidase GluC) and sequenced *de novo* by tandem mass spectrometry. In addition, the Edman degradation data of the intact protein allowed definition of the true N-terminus. The symbol ‘J’ is used to highlight the ambiguity between leucine and isoleucine in all positions other than the N-terminus, where Edman degradation was unambiguous. The positions of the disulfide bond are inferred by homology with similar proteins.

To gain further information about the 16 930 Da protein, a peptide sequencing strategy based on mass spectrometry was adopted. Two approaches were taken. First, Q-TOF tandem mass spectrometry of peptides obtained by direct infusion of proteolytic digests of the purified protein and secondly, LC–MS/MS of the peptide mixture on a second instrument that generated product ions at high mass accuracy and resolution. The sequencing strategy was based on digestion with three different endopeptidases (trypsin, endopeptidase LysC and endopeptidase GluC) to generate overlapping peptides that would cover as much of the primary sequence of the mature protein as possible, although unable to discriminate between the isobaric Leu/Ile pair, signified here by the residue ‘J’. In some instances, interpretation of the fragment ion mass spectra was assisted by labelling peptides using a 1 : 1 ratio of H_2_^16^O : H_2_^18^O in the digestion reaction. Only the **y**-series of ions, derived from the C-terminus of each peptide, are isotopically labelled in this reaction, and the doublets thus facilitated discrimination of the **b**- and **y**-ion series. Following interpretation of the amino acid sequence from the fragmented peptide, the theoretical *m/z* value of the [M+H]^+^ peptide was calculated and reconciled with the ions observed by MALDI-ToF. The complete sequence strategy is presented in figure 2, and the relevant peptide mass spectra are presented in the electronic supplementary material.

Edman degradation predicted an N-terminal tryptic peptide (HSEIDEK) with a theoretical [M+H]^+^ mass of *m/z* = 857.4. No peptide was detected at [M+H]^+^ 857.4 Da in either MALDI-ToF MS analysis of trypsin or LysC peptides. However, fragmentation of the tryptic peptide at [M+2H]^2+^ *m/z* = 393.21 yielded the sequence HSEJDGK (figure 2, peptide t1). This sequence included the highly conserved glycine residue of the N-terminal lipocalin motif (GXW), aligned with the ambiguous G/E call from the Edman sequencing confirming a glycine residue at this position. The second tryptic peptide within the Edman sequence was predicted as [M+2H]^2+^, *m/z* = 700.9; the sequence was determined as WVTVAJAADNVNK (t2) from the **b**- and **y**-ion series using ^18^O labelling; this contained the tryptophan residue of the GXW conserved motif. The N-terminal region was extended by tandem MS of a miscleaved peptide [M+3H]^3+^, *m/z* = 748.08 as WVTVAJAADNVNKJEEGGSJR (t3), also present at [M+H]^+^ *m/z* = 2242.13 in MALDI-ToF MS analysis of tryptic peptides. The sequence of the [M+H]^+^ 2242.13 tryptic peptide was confirmed by the [M+2H]^2+^ 430.7 tryptic peptide t4 (JEEGGSJR).

Since a feature of OBP-like proteins is the presence of two conserved disulfide bonds, the positions of cysteine residues were identified by carbamidomethylation. MALDI-ToF analysis of tryptic peptides from non-reduced preparations identified two peptides at [M+H]^+^ *m/z* = 1137.51 and *m/z* = 2131.04 that were shifted upon carbamidomethylation to [M+H]^+^ *m/z* = 1253.56 and *m/z* = 2247.10, a Δmass of 116 Da. The sequence of the reduced and alkylated peptide [M+H]^+^ *m/z* = 1253.56, isolated on LC–MS as the [M+2H]^2+^ *m/z* = 627.25 (T5), was EJTC*TEAC*NR, containing two modified cysteine residues. The Δmass of 116 Da following reduction and alkylation could not be explained simply by the carbamidomethylation of the two cysteine residues, which would generate a Δmass of 114.032 Da (2 × 57.016 Da). The additional 2 Da difference is explained by the reduction of a disulfide bond formed between the two cysteine residues. Since the unmodified peptide [M+H]^+^ *m/z* = 1137.51 is detected in oxidizing conditions, neither cysteine residue could have formed a disulfide bond with a second cysteine residue from a different region of the protein. Furthermore, a high-resolution peptide T6 [M + 2H]^2+^ *m/z* = 842.92 sequenced as JYJREJTCTEAC*NR. A tight disulfide loop separated by three amino acids is also a feature of other lipocalins and OBPs, including aphrodisin [57]; this provided further presumptive evidence that this protein is an aphrodisin-like lipocalin.

Using similar logic and further tandem MS, the entire sequence of the protein was recovered. All high-resolution peptide tandem mass spectra and sequence calls are provided in the electronic supplementary material. The protein sequence predicted a total length of 149 amino acids. The predicted average mass of the protein was 16 934 Da, which, when adjusted to 16 930 Da to allow for the loss of 4 Da through formation of the disulfide bonds at C_36_–C_40_ (proved) and C_55_–C_147_ (surmised, but consistent with homology modelling), correctly predicted the intact mass measured for the urinary protein.

**Figure 3.**
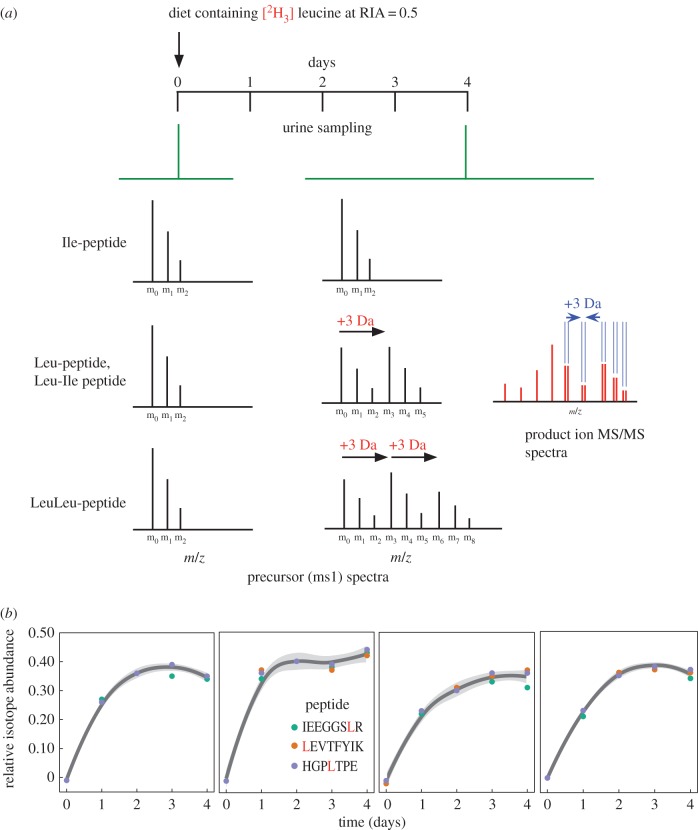
Metabolic labelling strategy to discern leucine and isoleucine residues. (*a*) Bank voles were fed a diet containing [^2^H_3_] leucine at a relative isotope distribution of 0.5. Incorporation of stable isotope-labelled leucine into peptides permits discrimination between leu and ile residues, either from precursor or product ion mass spectra. (*b*) After 4 days of labelling, urinary proteins (three representative peptides are show here for four voles) were labelled to the same extent as the dietary precursor and used for sequencing *de novo*.

Mass spectrometry-based sequencing *de novo* cannot distinguish between the isobaric amino acids leucine and isoleucine. To discriminate between this isobaric pair, voles were fed a diet partially labelled (relative isotope abundance of approx. 0.5) with [^2^H_3_]leucine. Because the protein was secreted in the urine, we surmised that the incorporation of this essential amino acid would result in specific labelling of leucine residues in the protein and in peptides derived therefrom. Both leucine and isoleucine are essential amino acids, and there is no mammalian metabolic pathway whereby the labelling centres in leucine could be transferred to isoleucine. After digestion with trypsin or endopeptidase Glu-C, or a double digest using both endopeptidases, partial labelling meant that each peptide (of monoisotopic mass M) containing a single leucine residue would be accompanied by a second mass, 3 Da heavier, leading to an M, M+3 Da doublet in both precursor and product ion spectra. Peptides containing solely isoleucine residues would not show any labelling doublet. Finally, peptides containing more than one leucine/isoleucine residue would require further analysis to locate the position of the leucine residues. The strategy is illustrated in figure 3, together with labelling profiles for several urinary glareosin peptides.

**Figure 4.**
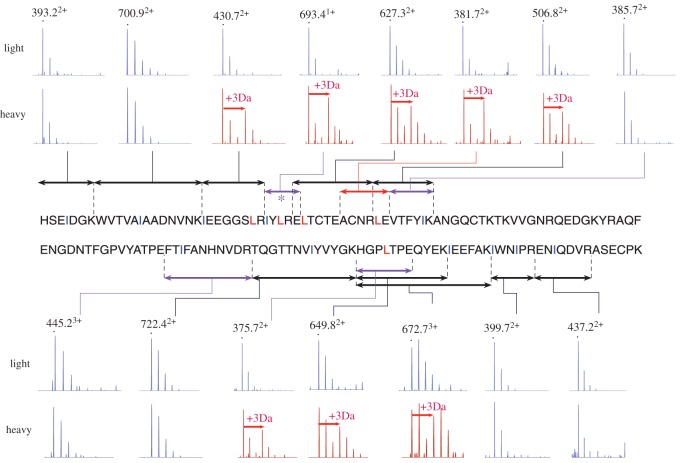
Resolution of leucine and isoleucine by metabolic labelling. After dietary administration of [^2^H_3_] leucine, proteolysis and mass spectrometry of the bank vole lipocalin, the assignment of leucine and isoleucine residues was completed. The figure indicates the residue assignment annotated with the precursor mass spectrum of the appropriate peptide (double-headed arrows), generated from trypsin (black), endopeptidase GluC (red) or digests using both endopeptidases (purple). The monoisotopic, unlabelled ion is marked with a black dot. Spectra that confirm the incorporation of a stable isotope-labelled leucine residue are coloured red and the mass offset (3 Da, due to incorporation of labelled leucine) is indicated with a red arrow.

All leucine/isoleucine ambiguities were evaluated manually and assigned from the raw data (figure 4). Tryptic peptides containing a single ambiguous site (defined as ‘J’; HSEIDGK isobaric identity known from Edman degradation, WVTVAJAADNVNK, EJTCTEACNR, TQGTTNVJYVYGK, HGPJTPEQYEK, ENJQDVR, ACNRJLE, VTFYJK, FTJFANHNVDR) were readily resolved from the precursor ion spectra. For peptides that contained more than a single instance of leucine or isoleucine, the strategy was more complicated. Most simply, precursor ion spectra could disambiguate peptides that contained two of the same residues (JWNJIPR), as the mass shift was unambiguous (+6 Da, Leu/Leu; 0 Da, Ile/Ile). For peptides that contained two Leu/Ile residues, only one of which was labelled, the precursor ion mass shift indicated the number but not the position of the leucine and isoleucine residues. Positional resolution was achieved by inspection of fragment ion spectra (electronic supplementary material). Fragment ion spectra were examined for+3 Da increases in the **y**- and **b**-ion series at each Leu/Ile product ion, clearly flagged as a doublet. This defined the position of Leu and Ile for peptides JEEGGSJR, JYJRE and JVTFYJK (electronic supplementary material). The remaining unassigned Leu/Ile site was in the small tryptic peptide JEEFAK (t13; [M+H]^+^= 736.3875 *m/z*), which is identical to an equivalent tryptic peptide derived from OBP2 and OBP3 [31]. To resolve this issue, we assigned the residue identity using tryptic missed cleavage peptides (this work: HGPLTPEQYEKJEEFAK compared to the peptide GQPLTPEQYEKLEEFAK from OBP2 and OBP3 (Uniprot D3VW63_MYOGA and D3VW64_MYOGA), respectively). The first leucine residue for the protein described here had already been confirmed (see previously) and the precursor mass spectrum of the missed cleavage peptide had a fragment ion distribution consonant with one leucine residue and one isoleucine residue, whereas the OBP peptide also present in LC–MS/MS analysis displayed a fragment isotopic distribution consistent with the presence of two heavy leucine residues (data not shown).

**Figure 5.**
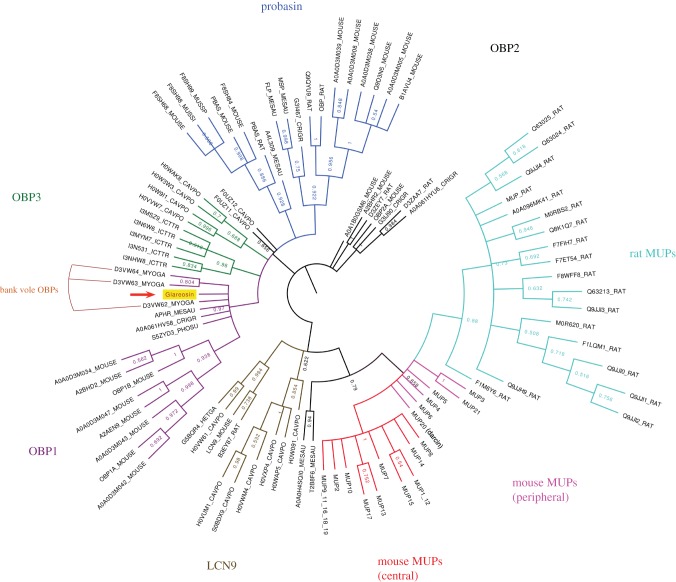
Phylogenetic tree of glareosin-related sequences. Bootstrapped maximum-likelihood phylogenetic tree calculated using MEGA7 as described in Material and methods. Branches corresponding to partitions reproduced in less than 50% bootstrap replicates were collapsed. With the exception of a manually curated set of mouse MUPs based on the MGI database (http://www.informatics.jax.org/searchtool/Search.do?query=mup*), proteins are labelled with UniProt identifiers. The three OBPs previously identified in bank voles [31] are highlighted.

We were thus able to derive the complete, unambiguous sequence of the bank vole urinary protein, including the identification of all leucine and isoleucine residues. The entire sequence was used in a BLAST search against all rodent sequences. The first major conclusion is that this abundant protein in bank vole urine is novel, and has not been reported previously. To distinguish this protein from other bank vole urinary proteins [31], we therefore propose the name ‘glareosin’ (derived from the species *M. glareolus*). The glareosin sequence matched to several lipocalins, most strongly to aphrodisins and OBPs, with weaker matches to probasins (prostate expressed ‘outlier’ lipocalins) and MUPs. A phylogenetic tree (figure 5) defines the relationships between these groups of lipocalins, specifically those from rodents, and a full alignment is given in the electronic supplementary material.

Of interest is the relationship between glareosin and the OBPs (1a, 1b, 2 and 3) that have previously been detected in bank vole urine samples [31]. The four proteins share over 60% sequence identity, and the presence of the lipocalin GXW motif and the disposition of the two disulfide bonds mean that all four proteins share a high level of structural and possibly functional similarity. Yet glareosin was not discovered or described as the predominant urinary protein in the previous study [31], in which the two urinary proteins detected on two-dimensional electrophoresis followed by mass spectrometry were OBP2 and OBP3. Indeed, when we perform a discovery proteomics analysis on a tryptic digest of total urinary proteins, we also see good evidence for these two proteins in bank vole urine (data not shown) but at a much lower level than peptides derived from glareosin. On a one-dimensional SDS–PAGE gel, glareosin is by far the most strongly expressed protein, and at first glance it is not obvious why this protein was not observed in the previous study. However, analysis of the sequence of glareosin and the three OBPs reveals that the predicted isoelectric point (pI) of OBPs 1–3 are 5.0, 4.8 and 4.8, respectively. By contrast, the predicted pI of glareosin is 5.7. In the previous study [31], the pI range of the two-dimensional gel system used to visualize and identify urinary proteins was from 3.9 to 5.1. It is highly likely that glareosin was not resolved by the first, isoelectric focusing dimension, would not have entered the gel and thus could not have been detected.

The complete protein sequence derived by mass spectrometry, including disambiguation of leucine/isoleucine, allowed us to submit the primary sequence to three-dimensional structure prediction. Of predicted structures for glareosin, those produced with a single alignment to aphrodisin [58–60] consistently scored better than those produced with either the top five or top 10 templates identified by HHpred. Aphrodisin is distinctly more closely related to glareosin (47% sequence identity) than other templates (39% at most)—this reinforces the observation that inclusion of more distantly related templates does not always benefit model quality when a closely homologous structure is available. Models generated with the initial HHpred alignment of glareosin with aphrodisin consistently exhibited stereochemical problems near the C-terminus where glareosin has a one-residue deletion compared to aphrodisin. Examination of the aphrodisin structure suggested that side chain interactions would be better retained with a one-residue shift of the deletion position. Positioning the deletion opposite Thr_149_ (mature protein sequence) in the aphrodisin template eliminated serious stereochemical issues and produced better scoring models by validation metrics.

**Figure 6.**
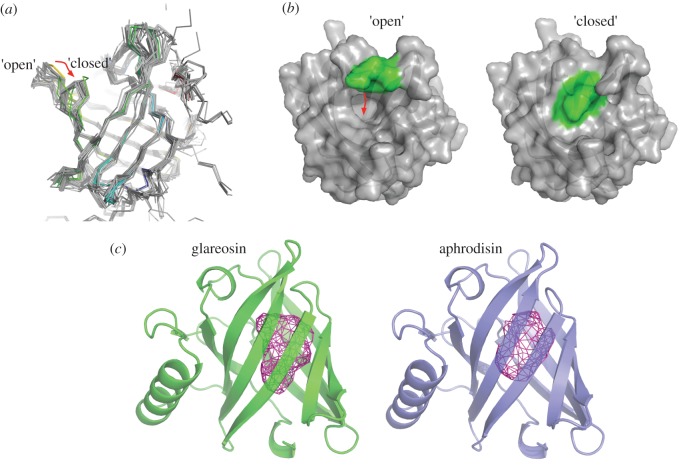
Predicted three-dimensional structure of glareosin. (*a*,*b*) The structure of glareosin was predicted by homology modelling. Two solutions (an ‘open’ and a ‘closed’ conformation) were predicted equally well. In (*a*), the two solutions are coloured blue to red from N- to C-terminus with all experimental structures of lipocalins sharing at least 25% sequence identity with glareosin shown in grey. In (*b*), the loop differing in conformation is shown as green, and the rest of the glareosin models as grey. (*c*) The cavity at the centre of the closed glareosin structure was analysed using the Profunc server [48] and compared with aphrodisin.

Unexpectedly, the final model set contained two distinct conformations which scored equally well by all criteria. Each conformation gives a normalized QMEAN *Z*-score of 0.44, showing that the structures, by the six distinct component scores considered, perform slightly better than the average protein of a similar size. The two conformations differ in the position of loop 5, connecting β-strands E and F (in the standard family nomenclature [56]). In the ‘closed’ conformation, the loop lies over the entry to the central binding pocket, as is typically observed in crystal structures (figure 6*a*), while in the ‘open’ conformation the entrance to the binding pocket is unimpeded and the pocket connects directly with bulk solvent. The validity of the two conformations is supported by the ability of the Rosetta methodology to accurately sample alternative, biologically relevant conformations: it has proved capable of predicting a second allosteric state accurately, given a crystal structure of the first [61]. Pathways of interconversion between these two conformational states could be explored in the future by molecular dynamics simulations. Interestingly, this loop bears a unique one-residue insertion compared to all near relatives of known structure. Thus, it is possible that glareosin has distinct ligand-binding properties when compared to other semiochemical lipocalins whose crystal structures, with cavity occupied or empty, show a strong tendency towards closed structures (figure 6*a*).

The central, β-barrel enclosed cavity of glareosin has a similar volume to aphrodisin; GHECOM [47] estimates them as 305 and 318 Å^3^, respectively, while the volumes from Profunc are 357 and 377 Å^3^. The cavity of the model structure of glareosin is more elongated, hinting at possible differences in specificity of bound ligands (figure 6*c*). For comparison, GHECOM predicts a cavity of 324 Å^3^ and Profunc 410 Å^3^ for the unoccupied MUP (1I04.PDB), and GHECOM 396 Å^3^ and Profunc 450 Å^3^ for a cavity occupied MUP (1I04.PDB). The glareosin cavity is thus of lower volume than the MUP, but is still large enough to accommodate a broad range of low-molecular weight ligands. Ligands of glareosin have yet to be identified.

It has previously been reported that the urinary protein output of *M. glareolus* is sexually dimorphic and that males exhibited obligate proteinuria in all sample types investigated [24]. Males mark new territory in frequent small drops without entirely emptying their bladder, compared with females that deposit large pools of urine [27,28]. This sex-specific behaviour is similar to that of the house mouse, where the repeated marking of territory with small volumes of urine is used to advertise competitive dominance [62,63].

Glareosin appears to be the major protein output in male bank vole urine that is stimulated during the breeding season. As a lipocalin with a clearly defined central cavity that could be switchably accessible, combined with male-specific production and a seasonal expression pattern, this points to a role for glareosin as a major driver of chemical communication between male and female bank voles. As we gain a better understanding of the use of lipocalins in chemical communication in rodents, an interesting bifurcation is increasingly evident. Of rodents, Muridae (Old World mice, rats) have evolved polymorphic families of MUPs that create considerable potential for individual variation in proteins—they function as pheromone-binding proteins but also as pheromones in their own right. Currently, our knowledge is largely derived from studies of house mice (*M. musculus*) and brown rats (*Rattus norvegicus*). By contrast, Cricietinae (hamsters, voles) also elaborate protein in their secretions, but evidence thus far suggests that this is restricted to high levels of a single protein. Thus, roborovskin, from *Phodopus roborovskii*, is a single lipocalin produced in the urine equally by both sexes [64]. The vaginal discharge of the golden hamster, *Mesocricetus auratus*, contains abundant levels of the lipocalin aphrodisin, which acts as a pheromone (possibly in concert with a bound ligand) to stimulate copulatory behaviour by males [8,57,58,60,65]. Aphrodisin is a female-specific lipocalin in vaginal secretions, whereas glareosin is a male-specific protein restricted to the breeding season. While none of these species invoke the same polymorphic variation as MUPs as the Muridae, it is probable that clear functions in intraspecific communication will be found. Interestingly, Bathyerginae (*Fukomys*, naked mole rat) also seem to express urinary proteins that are more aphrodisin-like [66]. It is possible that MUP-like sequences have evolved different roles to aphrodisin/OBP-like proteins, and that in muroid rodents, a high level of polymorphism may be a unique feature. Whereas MUPs are readily identified and classified within the lipocalin family, there is a need for clearer understanding of the aphrodisin-like proteins. OBPs are expressed in nasal tissue in a wide range of species [67–72] and may facilitate the transport of low-molecular-weight signalling molecules across the mucosal membrane. However, OBPs are now being increasingly reported in the urine of rodents, and it is likely that they are also involved in the generation as well as the reception of chemosignals. Further study of the role of lipocalins in chemical communication seems likely to reveal a breadth of mechanisms whereby information is conveyed between conspecifics.

## Supplementary Material

Supplementary Methods S1; Supplementary Figure S2; Supplementary Figure S3; Supplementary Figure S4; Supplementary Figure S5

## Data accessibility

Detailed methods are presented in the electronic supplementary material. The mass spectrometry proteomics data have been deposited to the ProteomeXchange Consortium via the PRIDE [32] partner repository with the dataset identifier PXD006645 and 10.6019/PXD006645.
