# Supplementary material for: Glareosin: a novel sexually dimorphic urinary lipocalin in the bank vole, Myodes glareolus
Source: Open Biol. 2017 Sep 6;7(9):170135. doi: 10.1098/rsob.170135 (PMC5627053; doi:10.1098/rsob.170135)

Supplementary material

for

Grace M Loxley, Jennifer Unsworth, Michael J. Turton, Alexandra Jebb, Kathryn S Lilley, Deborah M Simpson, Daniel J. Rigden, Jane L. Hurst & Robert J. Beynon.(2017) Glareosin: a novel sexually dimorphic urinary lipocalin in the bank vole, *Myodes glareolus* Open Biology DOI: 10.1098/rsob.20160135

S1. Expanded Materials and Methods

Sampling: Samples from voles caught from two different geographic areas of the United Kingdom, the Leahurst campus in Merseyside and Kielder in Northumberland. Three different samples types were examined to evaluate any difference observed between cage deposited urine, urine removed directly from the bladder of an anaesthetised vole and urine possibly contaminated with preputial secretions since preputial secretions from *M. glareolus* have previously been implicated in displays of male dominance. Urine samples were collected from both wild caught and captive bred male *M. glareolus.*

Urine samples that were naturally expressed when the animal was handled were used for initial analysis of urinary proteins by SDS PAGE. All further experiments used cage deposited samples due to the low volume of naturally expressed urine (typically less than 5 µl). Deposited samples were collected from individual voles in cages previously cleaned rigorously by hand with dilute detergent, followed by four successive pure water rinses. Animals were placed in dry cages under the same laboratory conditions for 4 h with food and water provided *ad libitum*. Cage deposited urine samples were recovered by three successive 5 ml washes of Milli-Q purified water. The total protein concentration of each sample was calculated using the Coomassie Plus® protein assay (Pierce, Rockford, USA). Urine dilution was assessed by the total creatinine concentration of each sample, measured using the Sigma Diagnostics Creatinine Assay (Sigma, Poole, UK).

SDS-PAGE analysis: SDS-PAGE analysis of the urinary proteins of *M. glareolus* used a Tris-chloride/Tris glycine discontinuous buffer system under reducing conditions. Electrophoresis was conducted in a mini-protean system (Bio-Rad, Hemel Hempstead, UK) and separated proteins were visualized with Coomassie blue.

Anion exchange chromatography: Proteins from cage deposited samples were further purified by anion exchange chromatography using a UNO-Q anion exchange column (Bio-Rad, Hemel Hempstead, UK) equilibrated in 10 mM HEPES pH 8.0 and were eluted with a 0 - 0.5 M NaCl gradient at 1ml/min. The absorbance of the eluate was measured at 280 nm and 0.5 ml fractions were collected. The fractions were subsequently analysed by SDS PAGE and electrospray ionisation mass spectrometry (ESI-MS).

In-gel proteolysis: Protein bands from SDS-PAGE were digested with trypsin to generate peptides suitable for further analysis by MALDI-ToF mass spectrometry. Excised gel plugs (approx. 1mm^3) were destained with 50 µl of

50 mM NH_4HCO_3/50 % (v/v) acetonitrile for 15 min. They were then reduced and carbamidomethylated by the addition of 50µl of 10mM dithiothreitol and 50µl of 55 mM iodoacetamide for 20 min. Following reduction and alkylation the gel plug was washed with 50µl 100 mM NH_4HCO_3 for 10 min and dehydrated with 50 µl of acetonitrile for a further 10 min. Excess acetonitrile was removed and the dry gel plug was rehydrated with 2 µl of 6 ng/µl sequencing grade trypsin (Roche, Lewes, UK) in 50 mM NH_4HCO_3, pH 8.5. The rehydrated gel plug was then incubated at 37ºC for 5 h following which the remaining supernatant was removed and combined with peptides that were extracted from the gel plug by the addition of 9 µl of 1% (v/v) formic acid/2 % (v/v) acetonitrile for 15 min.

Edman degradation: SDS PAGE gels before staining were electroblotted to polyvinylidene difluoride (PVDF) membranes for N-terminal sequencing using an Applied Biosystems 476A gas phase sequencer (Applied Biosystems). After electroblotting, the PVDF was stained with Coomassie blue to visualize protein bands prior to excision and Edman degradation.

In-solution proteolysis: Aliquots of protein (10ug) purified from cage deposits by anion exchange chromatography were precipitated with a final concentration of 30 % (w/v) trichloroacetic acid (TCA) and washed three times in ether. To reduce disulphide bonds, the pellet was resuspended in 100 µl of 20 mM DTT in 50 mM NH_4HCO_3 and incubated at 55 °C for 1 hour. The sample was then carbamidomethylated with 100µl of 55 mM iodoacetamide in 50 mM NH_4HCO_3 and incubated at 37 °C for 1 hour in the dark. The product was again precipitated with a final concentration of 30% (w/v) TCA and resuspended in 50 mM NH_4HCO_3/pH8.5 containing 0.4 µg/µl of sequencing grade trypsin or endopeptidase Glu-C (Roche, Lewes, UK), prior to being incubated overnight at 37 °C. Digestion with endopeptidase Lys-C (Roche, Lewes, UK) followed a similar protocol using 25 mM Tris HCl, 1 mM EDTA pH 8.5 as the digestion buffer.

MALDI-ToF mass spectrometry: Analysis of peptides from in-gel digests was undertaken using a M@LDI-TOF reflectron mass spectrometer (Waters, Manchester, UK) in positive ion mode. The instrument was calibrated using a peptide mix (des-Arg-Bradykinin (2.4 pmol/µl 1,904.47 Da), neurotensin (2.4 pmol/µl, 1672.92 Da), adenocorticotropic hormone clip 18-39 (2.6 pmol/µl, 2465.20 Da) and oxidised insulin β-chain (30 pmol/µl, 3495.9 Da). Samples were prepared for analysis by co-crystalisation with an equal volume of matrix solution (saturated α-cyano-4-hydroxy cinnamic acid in a 1:1:1:1 (v/v) solution of acetonitrile:H_2O:methanol:0.1 % (v/v) TFA). A 1µl aliquot of the peptide/matrix mixture from each sample was deposited onto a 96 well MALDI target and allowed to dry at room temperature. Spectra were acquired between 1000 and 4000 m/z with the laser energy optimized to give the best signal to noise ratio for each sample. The laser firing rate was 5 Hz and 10 spectra (collected over 2 s) were combined. The final mass spectrum was a combination of 10-15 such combined data sets, representing 100-150 individual laser shots. Each spectrum was then internally recalibrated using the trypsin autolysis peak at 2163.057 Da or by the addition of an internal calibrant, neurotensin (1672.918 Da), (120 fmol/µl). All aspects of data acquisition, processing and machine management were controlled through the MassLynx software suite (version 4.0).

Electrospray ionization mass spectrometry: ESI-MS was used in two modes; liquid chromatography-mass spectrometry (LC-MS) was used for intact mass analysis whilst tandem mass spectrometry (MS/MS) was used for peptide sequence analysis. All ESI-MS was undertaken on a Q-ToF Micro mass spectrometer (Waters, Manchester, U.K.) in positive ion mode. The instrument was calibrated with the product ions of a 500 fmol/µl (Glu1)-fibrinopeptide B (GluFib) solution in 50% (v/v) acetonitrile/0.1% (v/v) formic acid, infused from a syringe pump at 0.5 µl/min through a PicoTip emitter (New Objective, Massachusetts, USA). For intact mass analysis the instrument was operated in ToF only mode. Samples were desalted on-line with a C4 reverse phase trap and subsequently introduced into the mass spectrometer in a solution of 90% (v/v) acetonitrile/0/1% (v/v) formic acid. Raw data were gathered between 700 and 1400 m/z at a scan/interscan time of 2.4 s/0.1 s. These raw data were subsequently de-convoluted using the MaxEnt 1 module contained within the MassLynx 4.0 software. For MS/MS analysis of proteolytic peptides, precursor spectra were acquired between 400 and 1500 Th at a scan/interscan time of 1.0 s /0.1 s. Product ion spectra were acquired between 100 and 2000 m/z at the same scan/interscan speed. Raw product ion spectra were deconvoluted using the MaxEnt 3 algorithm in the MassLynx software, with the charge state of the parent peptide determined from the isotope envelope in the precursor ion spectrum. Interpretation of product ion spectra and the determination of peptide sequences were facilitated by the PepSeq module within MassLynx 4.0. As an additional aid in the interpretation of tandem mass spectra, peptides were isotopically labelled with ^{18}O by performing proteolytic digestion in a 1:1 mix of light (H_2[^{16}O]) and heavy (H_2[^{18}O]) water. This allowed the incorporation of a 1:1 mixture of [^{16}O] and [^{18}O] atoms into the newly formed C-termini of peptides. Following tandem mass spectrometry, the y-ions were readily identified as a sequence of doublets of approximately equal intensity, separated by 2 Da. To confirm the sequence, we repeated the digestions and analysed the samples on a high-resolution instrument with high mass accuracy and resolution for precursor and product ions.

To collect data at high resolution, samples were analysed using a Ultimate 3000 nano system (Dionex/Thermo Fisher Scientific, Hemel Hempstead, UK) coupled to a QExactive mass spectrometer (Thermo Fisher Scientific, Hemel Hempstead, UK). Peptides (500 fmol) were loaded onto a trap column (Acclaim PepMap 100, 2cm x 75□m inner diameter, C18, 3 µm, 100Å) at 5 µL/min with an aqueous solution containing 0.1%(v/v) TFA and 2%(v/v) acetonitrile. After 3 min, the trap column was set in-line with an analytical column (Easy-Spray PepMap® RSLC 15cm x 75 µm inner diameter, C18, 2 µm, 100Å) (Dionex). Peptides were eluted by using an appropriate mixture of solvents A and B. Solvent A was HPLC grade water with 0.1%(v/v) formic acid, and solvent B was HPLC grade acetonitrile 80%(v/v) with 0.1%(v/v) formic acid. Separations were performed by applying a linear gradient of 3.8% to 50% solvent B over 35 min at 300nL/min followed by a washing step (5 min at 99% solvent B) and an equilibration step (15 min at 3.8% solvent B). The mass spectrometer was operated in data dependent positive (ESI+) mode to automatically switch between full scan MS and MS/MS acquisition. Survey full scan MS spectra (300-2000 m/z) were acquired in the Orbitrap with 70,000 resolution (200 m/z) after accumulation of ions to 1x106 target value based on predictive automatic gain control (AGC) values from the previous full scan. Dynamic exclusion was set to 20s. The 10 most intense multiply charged ions ($z \geq 2$) were sequentially isolated and

fragmented in the octopole collision cell by higher energy collisional dissociation (HCD) with a fixed injection time of 120ms and 35,000 resolution. The mass spectrometer was calibrated using a ready to use positive ion calibration solution from the instrument manufacturer (Thermo Fisher Scientific, Hemel Hempstead, UK). The solution contains a mixture of caffeine, MRFA, Ultramark 1621, and n-butylamine in an acetonitrile:methanol:water solution containing acetic acid (1% v/v). The mass spectrometer conditions were as follows: spray voltage, 1.9kV, no sheath or auxiliary gas flow; heated capillary temperature, 250; normalised HCD collision energy 30%. The MS/MS ion selection threshold was set to 1 x 10^4 counts and a 2 m/z isolation width was set.

5,5,5-d3-leucine labelled diet: To discriminate between isobaric leucine and isoleucine residues, we fed bank voles a diet containing stable isotope labelled leucine. Low-sugar rodent diet (100g, 1.16% w/w leucine) was suspended in milliQ-grade H_2O. [$^2H_3$]leucine (5,5,5-d3-leucine) was added (1.16g) to a level equivalent to that present in the diet in unlabeled form and the diet and label were blended thoroughly in a food processor. The diet was then dehydrated for 48 hours in a commercial food drier. Cage deposited urine samples were collected from four voles (day 0) before they were transferred to a new cage with the [$^2H_3$]leucine diet provided *ad libitum*. Cage deposited urine samples were then collected daily for four days. Unlabelled diet was resumed after the final urine samples were collected. Urine samples were then stored at -20°C until analysis. Urinary proteins were reduced, alkylated and digested with trypsin in solution, followed by LC-MS/MS analysis on the QExactive-HF (Thermo Scientific™) as described above. Leucine and isoleucine residues were then manually assigned from the raw data, and confirmed with MASCOT and PEAKS searches under the same search conditions as below with triple labelling with deuterium as an additional variable modification, against the derived sequence of glareosin.

Protein sequence analysis: The final amino acid sequence was used in a BLAST search (32) using default parameters for protein matches against Rodenta. The 138 matches were reduced and processed as follows. First, incomplete sequences, sequences substantially larger than the core lipocalin size of approx. 160 amino acids or those that only matched across part of the sequence were eliminated. Some sequence entries were exact duplicates and were reduced to single entries. Finally, because we wished to compare the glareosin secreted protein sequence, signal peptides were removed, either guided by the feature entry in the database entry or through the SignalP 4.1 server (33) (http://www.cbs.dtu.dk/services/SignalP/). The reduced sequence set was aligned with MAFFT using the high accuracy linsi algorithm (34) with Jalview (35) used to display and manipulate sequence alignments.

Phylogenetic analysis: The evolutionary history was inferred by using the Maximum Likelihood method based on the JTT matrix-based model (36). Bootstrapping analysis (37) using 500 replicates was carried out. Branches corresponding to partitions reproduced in less than 50% bootstrap replicates were collapsed. All positions containing gaps and missing data were eliminated leaving a total of 112 positions in the final dataset. Evolutionary analyses were conducted in MEGA7 (38).

Homology modelling: The structure of mature glareosin, without its signal peptide, was modelled using the Rosetta_CM protocol (39). Ten models were produced for each combination of templates and alignments. Templates were identified from a non-redundant library of PDB structures using the HHpred server (40) and modelling was done with one, five or ten templates assessing the results quantitatively with Rosetta's own energy function and with the Prosa II (41), DOPE (42) and QMEAN (43) protein structure quality metrics. Stereochemistry was assessed with PROCHECK (44). Structures were superimposed using GESAMT (45). Cavities were detected and measured using the GHECOM (46) and Profunc (47) servers. PyMOL (https://www.pymol.org/) was used to visualise and manipulate structures and to produce structure figures.

Supplementary material

Glareosin: a novel sexually dimorphic urinary lipocalin in the bank vole, *Myodes glareolus*

Grace M Loxley, Jennifer Unsworth, Michael J. Turton, Alexandra Jebb, Kathryn S Lilley, Deborah M Simpson, Daniel J. Rigden, Jane L. Hurst & Robert J. Beynon

S2. Full sequence coverage of glareosin

After the protein sequence was completed by sequencing *de novo*, multiple in-solution tryptic digests were prepared from different individuals, and the overall peptide coverage of the sequence was analysed using PEAKS. Peptides in blue are derived from the PEAKS-DB module, grey peptides are derived from PEAKS module SPIDER.

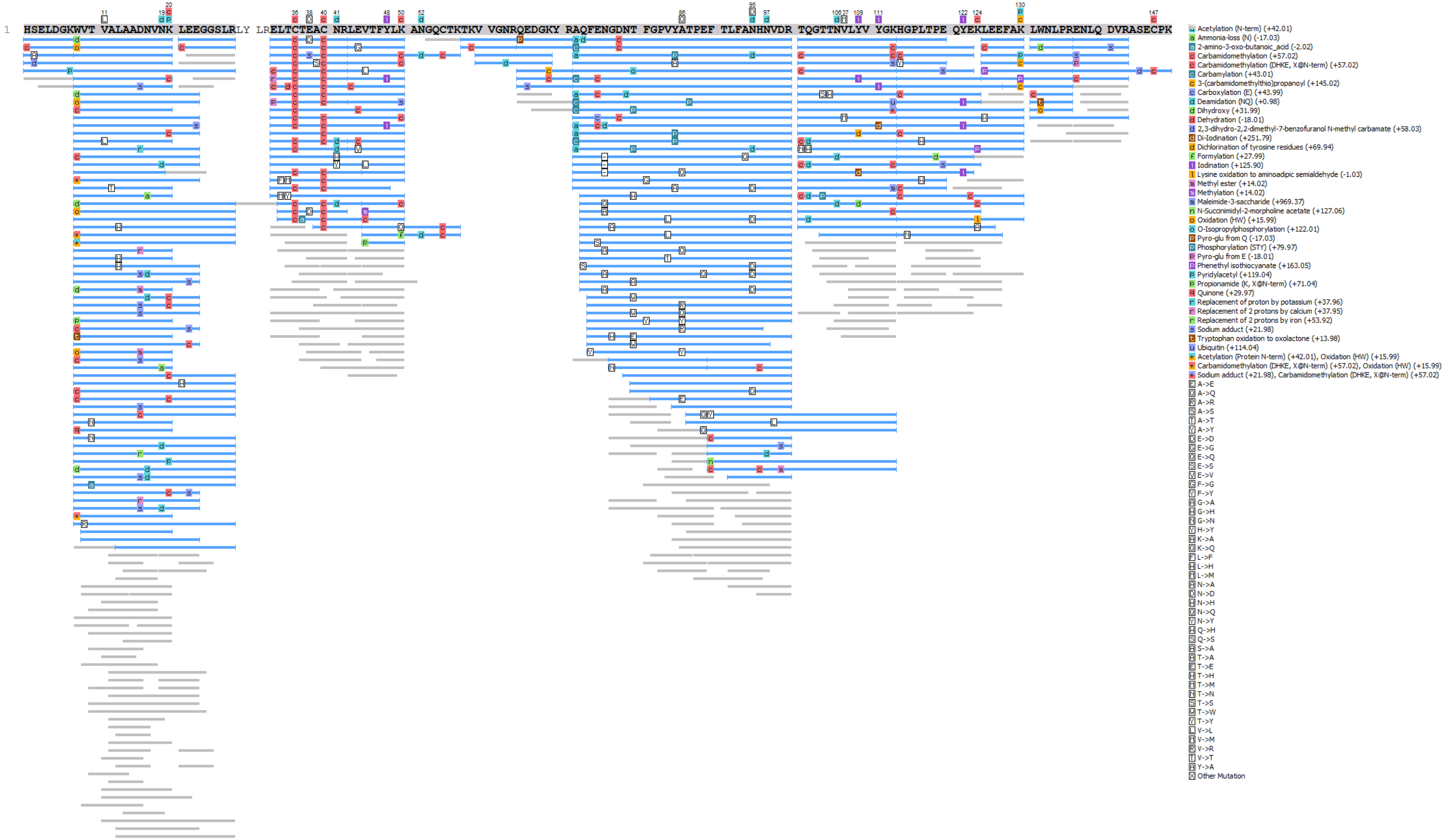

Supplementary material

Glareosin: a novel sexually dimorphic urinary lipocalin in the bank vole, *Myodes glareolus*

Grace M Loxley, Jennifer Unsworth, Michael J. Turton, Alexandra Jebb, Kathryn S Lilley, Deborah M Simpson, Daniel J. Rigden, Jane L. Hurst & Robert J. Beynon

S3. Sequencing of glareosin *de novo*

Full MS/MS data are shown for peptides derived from trypsin, endopeptidase LysC and endopeptidase GluC digests. Although all assignments are shown as leucine, this residue cannot be distinguished from the isobaric isoleucine residue, and should be taken as ambiguous in this sequencing. However, metabolic labelling with stable isotope labelled leucine was able to fully resolve these ambiguities.

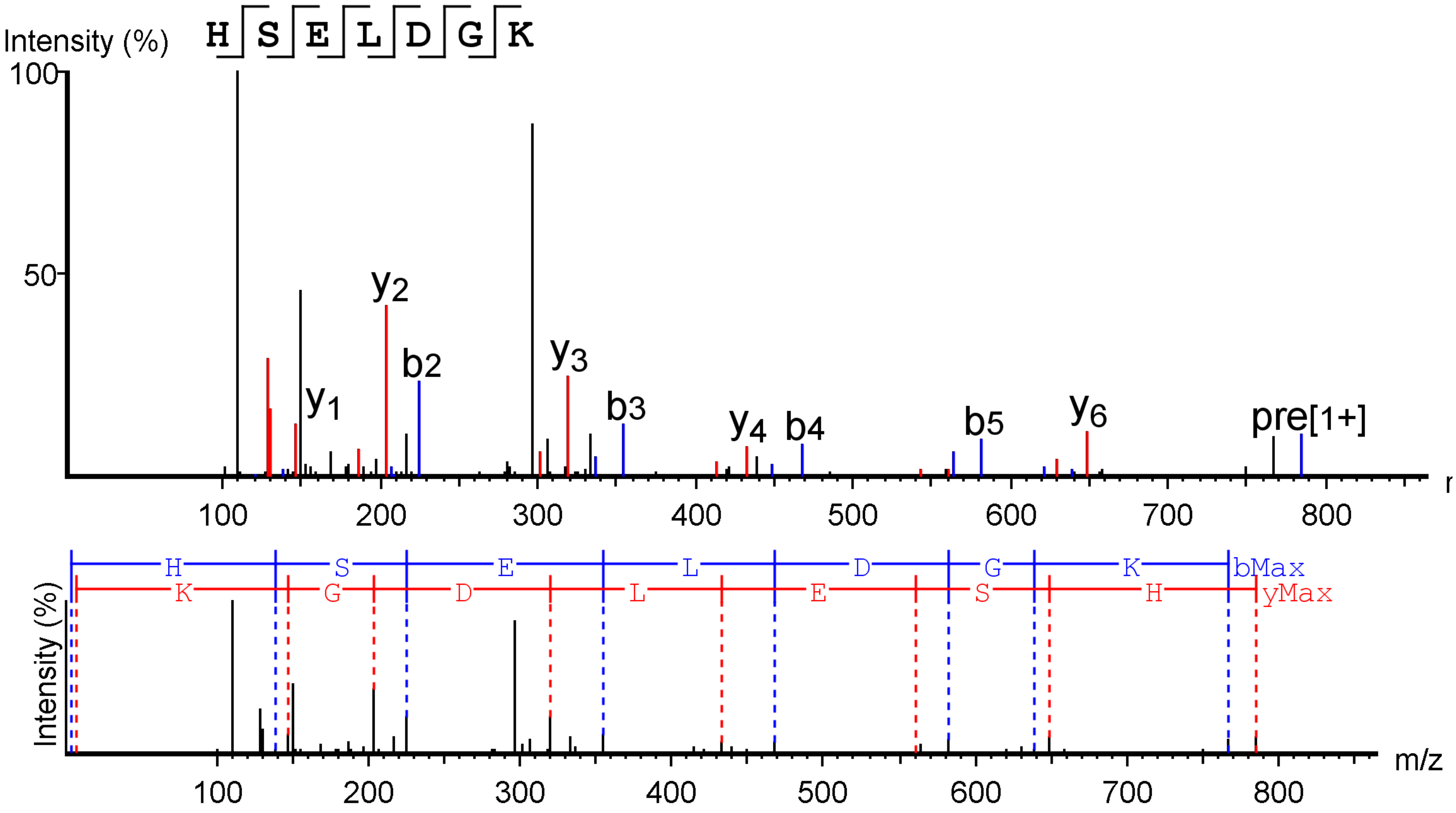

Figure 1. ***De novo*** **sequence analysis of the processed MS/MS spectra of** ***M. glareolus*** **tryptic peptide 785.3 m/z (Figure 2, t1)**

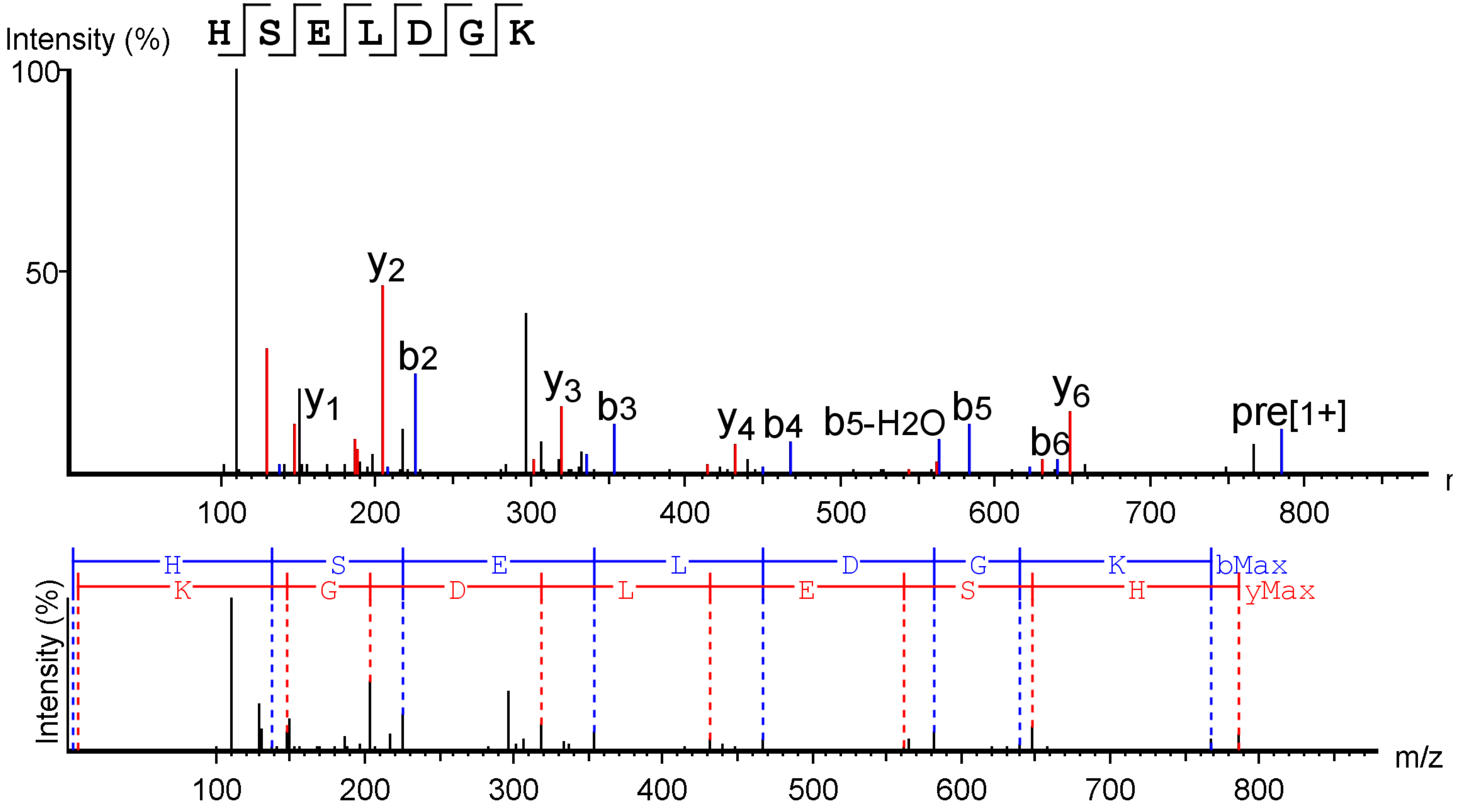

Figure 2. ***De novo*** **sequence analysis of the processed MS/MS spectra of** ***M. glareolus*** **LysC peptide 785 m/z (Figure 2, t1)**

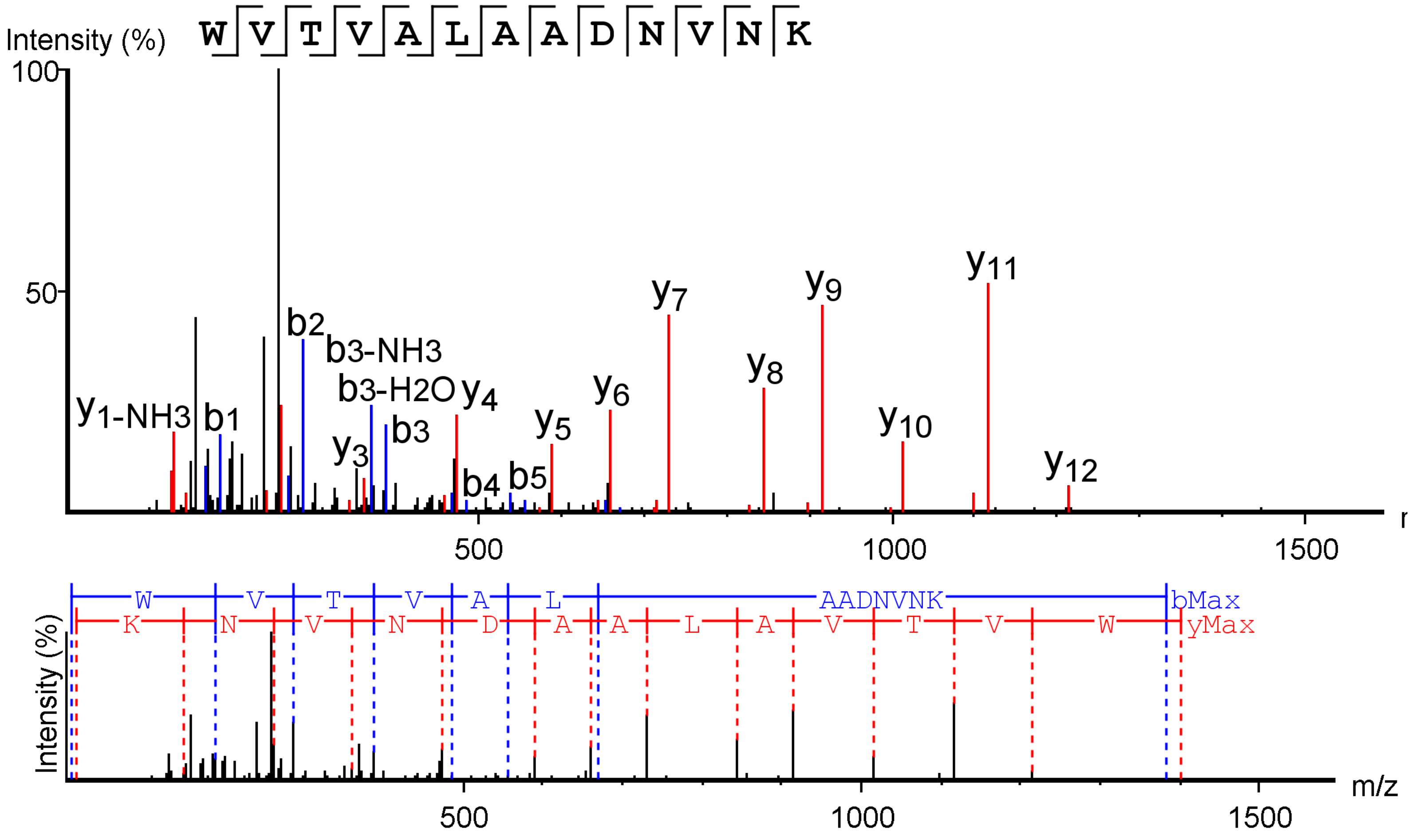

Figure 3 ***De novo*** **sequence analysis of the processed MS/MS spectra of** ***M. glareolus*** **tryptic peptide 1400.7 m/z (Figure 2, t2)**

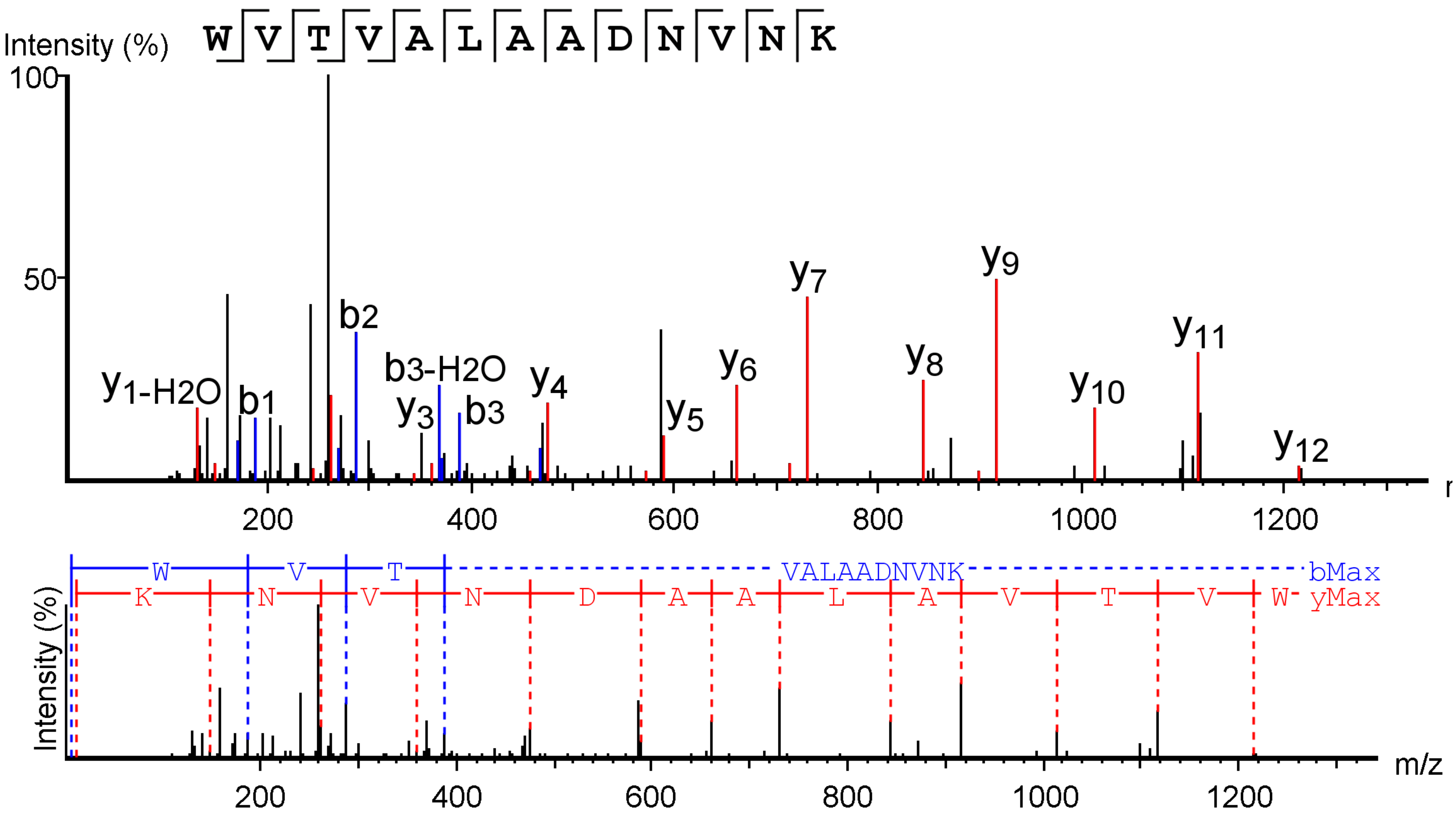

Figure 4. ***De novo*** **sequence analysis of the processed MS/MS spectra of** ***M. glareolus*** **LysC peptide 1400.7 m/z (Figure 2, t2)**

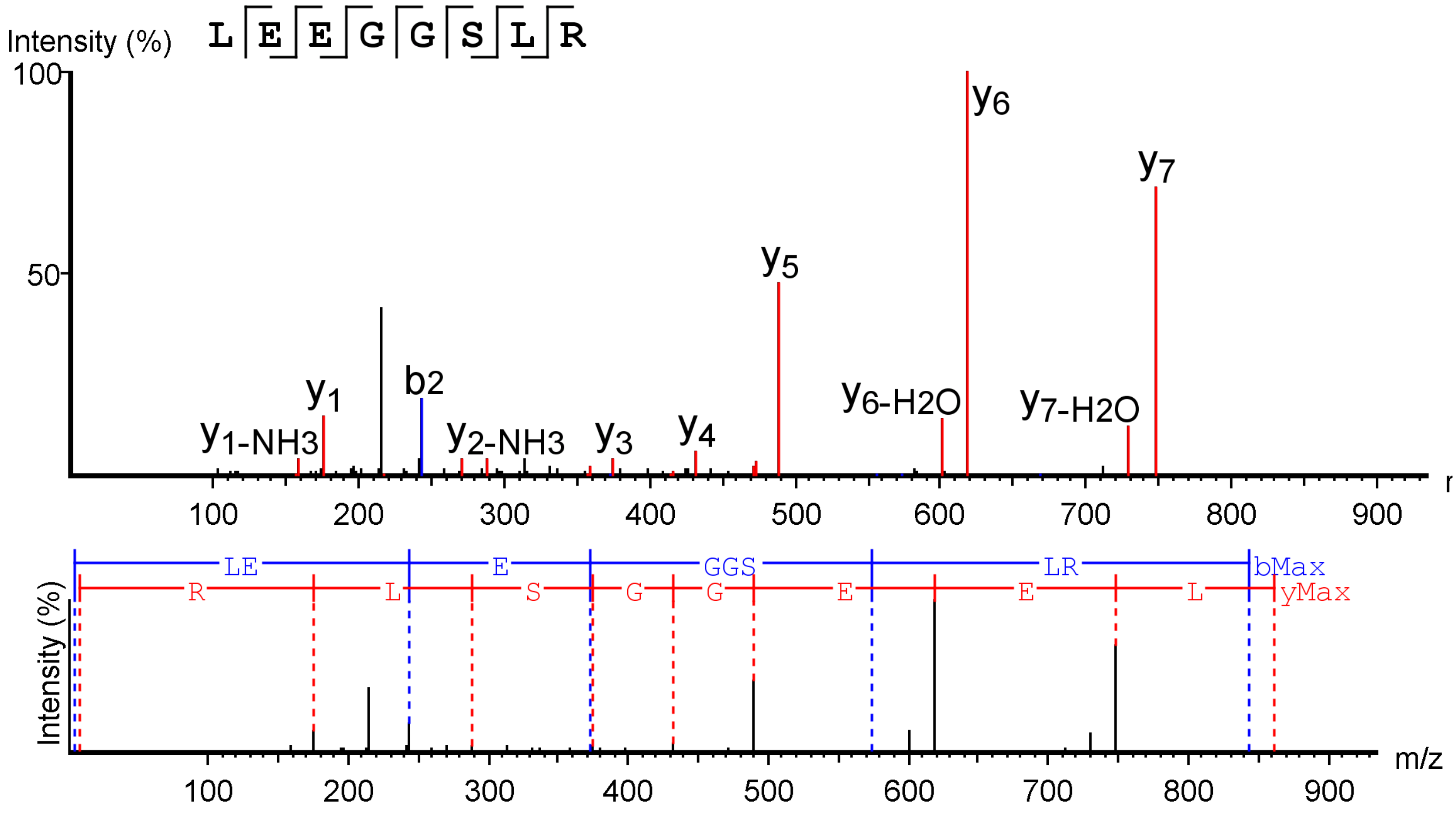

Figure 5. ***De novo*** **sequence analysis of the processed MS/MS spectra of** ***M. glareolus*** **tryptic peptide 860.4 m/z (Figure 2, t4)**

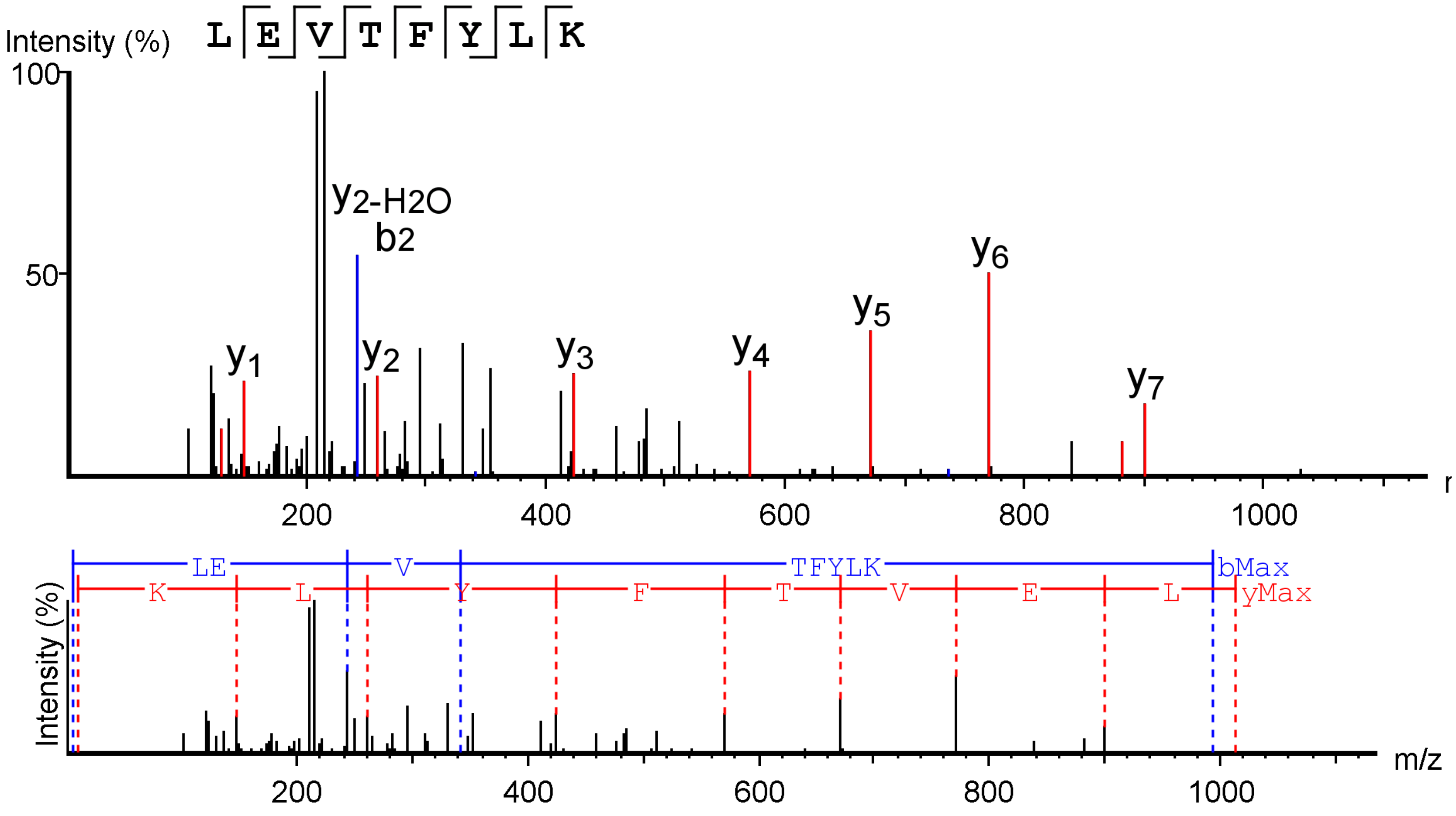

Figure 6. *De novo* **sequence analysis of the processed MS/MS spectra of** *M. glareolus* **tryptic peptide 1012.5 m/z (Figure 2, t8)**

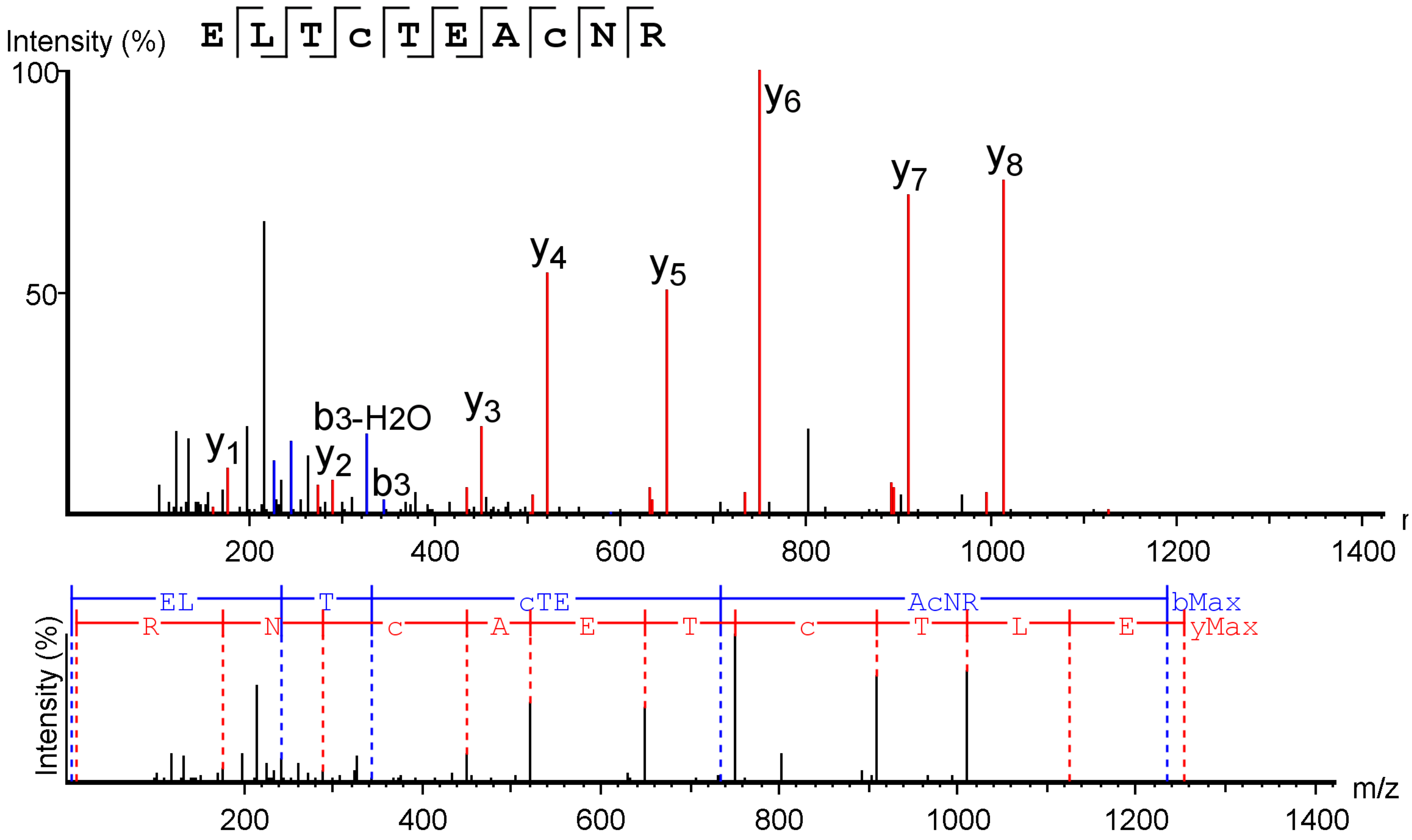

Figure 7. ***De novo*** **sequence analysis of the processed MS/MS spectra of** ***M. glareolus*** **tryptic peptide 1253.5 m/z (Figure 2, t5)**

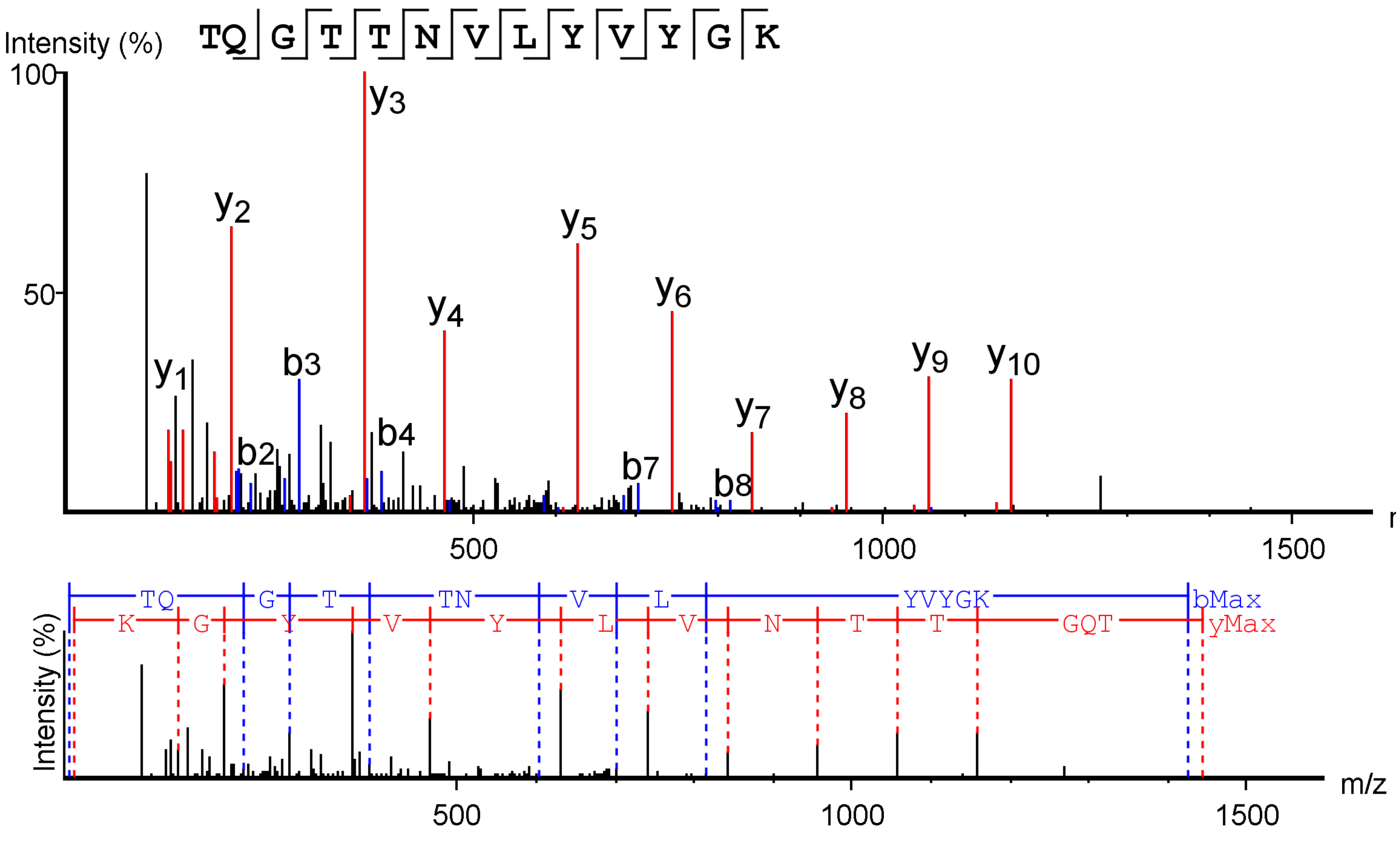

Figure 8. *De novo* **sequence analysis of the processed MS/MS spectra of** *M. glareolus* **tryptic peptide 1443.7 m/z (Figure 2, t11)**

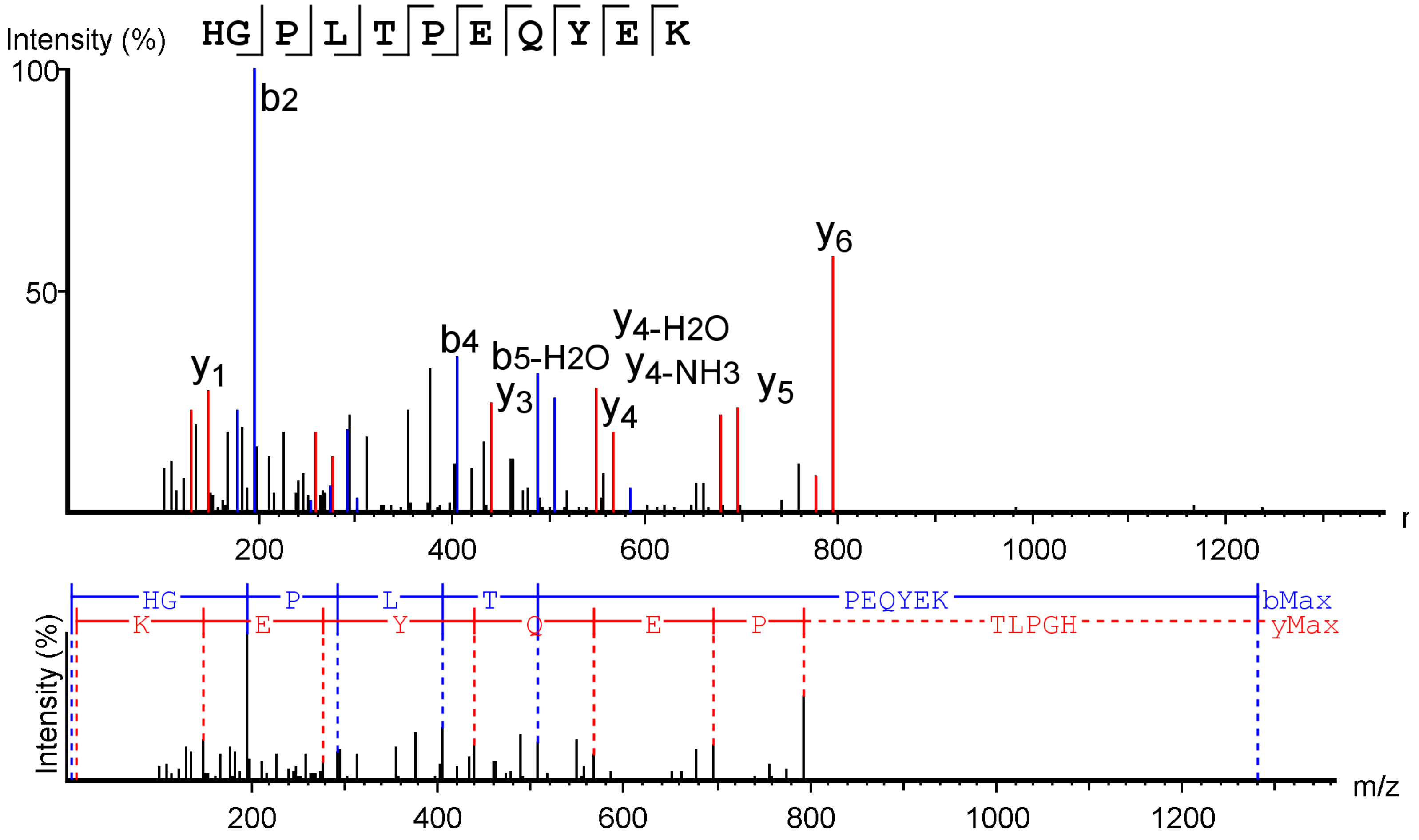

Figure 9. *De novo* **sequence analysis of the processed MS/MS spectra of** *M. glareolus* **tryptic peptide 1298.6 m/z(Figure 2, t12)**

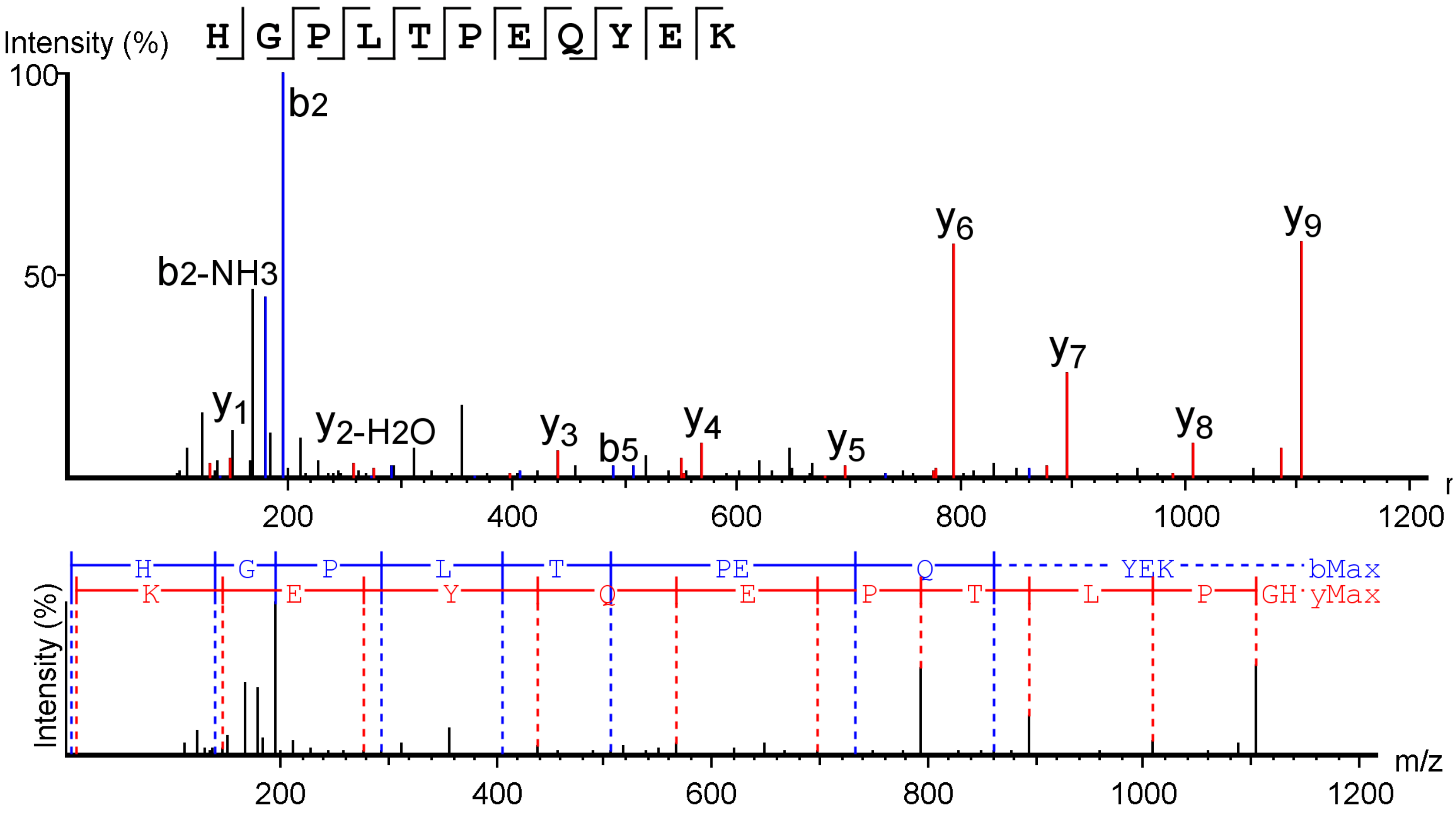

Figure 10. ***De novo*** **sequence analysis of the processed MS/MS spectra of** ***M. glareolus*** **LysC peptide 1298.6 m/z (Figure 2, t12)**

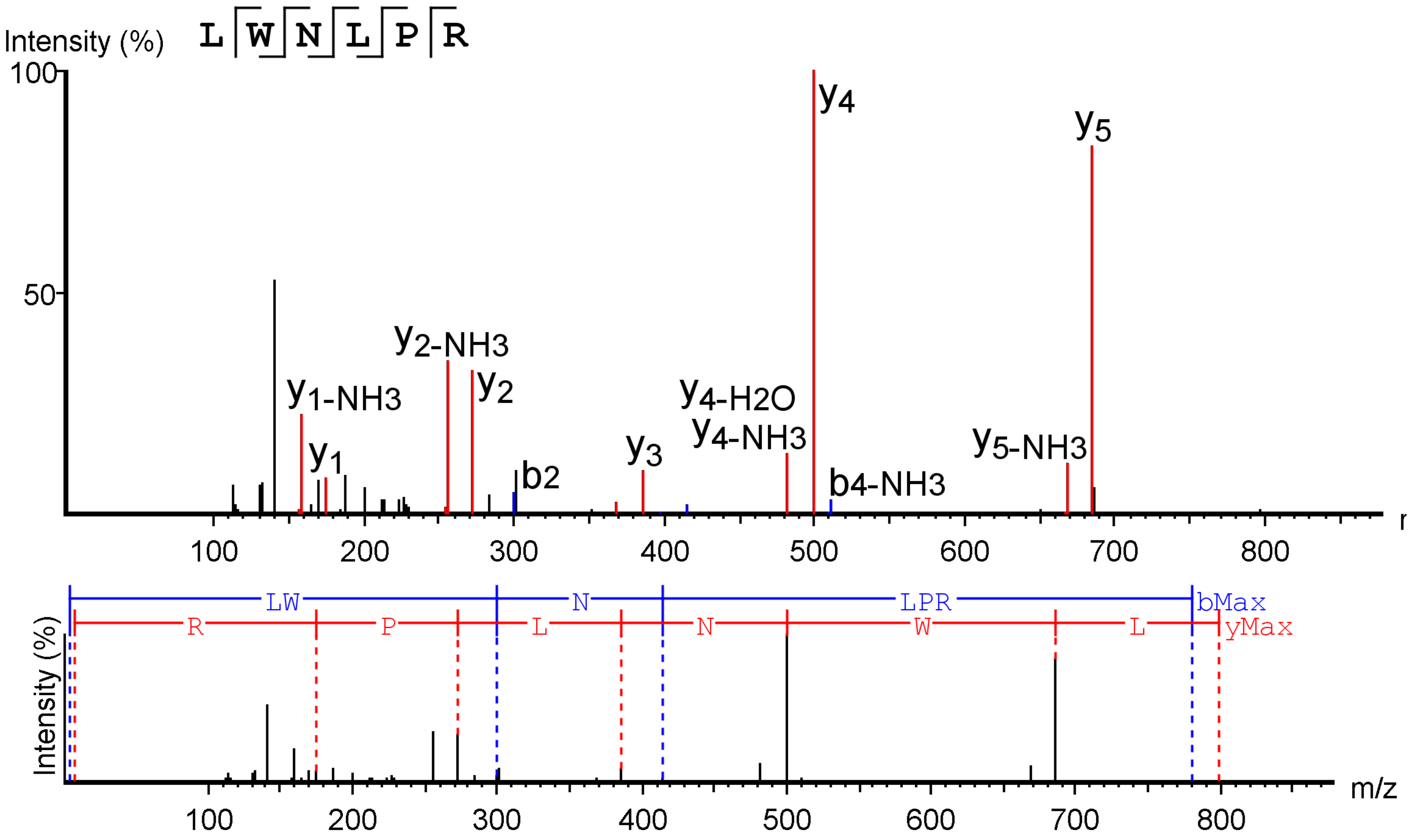

Figure 11. *De novo* **sequence analysis of the processed MS/MS spectra of** ***M. glareolus*** **tryptic peptide 798.4 m/z (Figure 2, t15)**

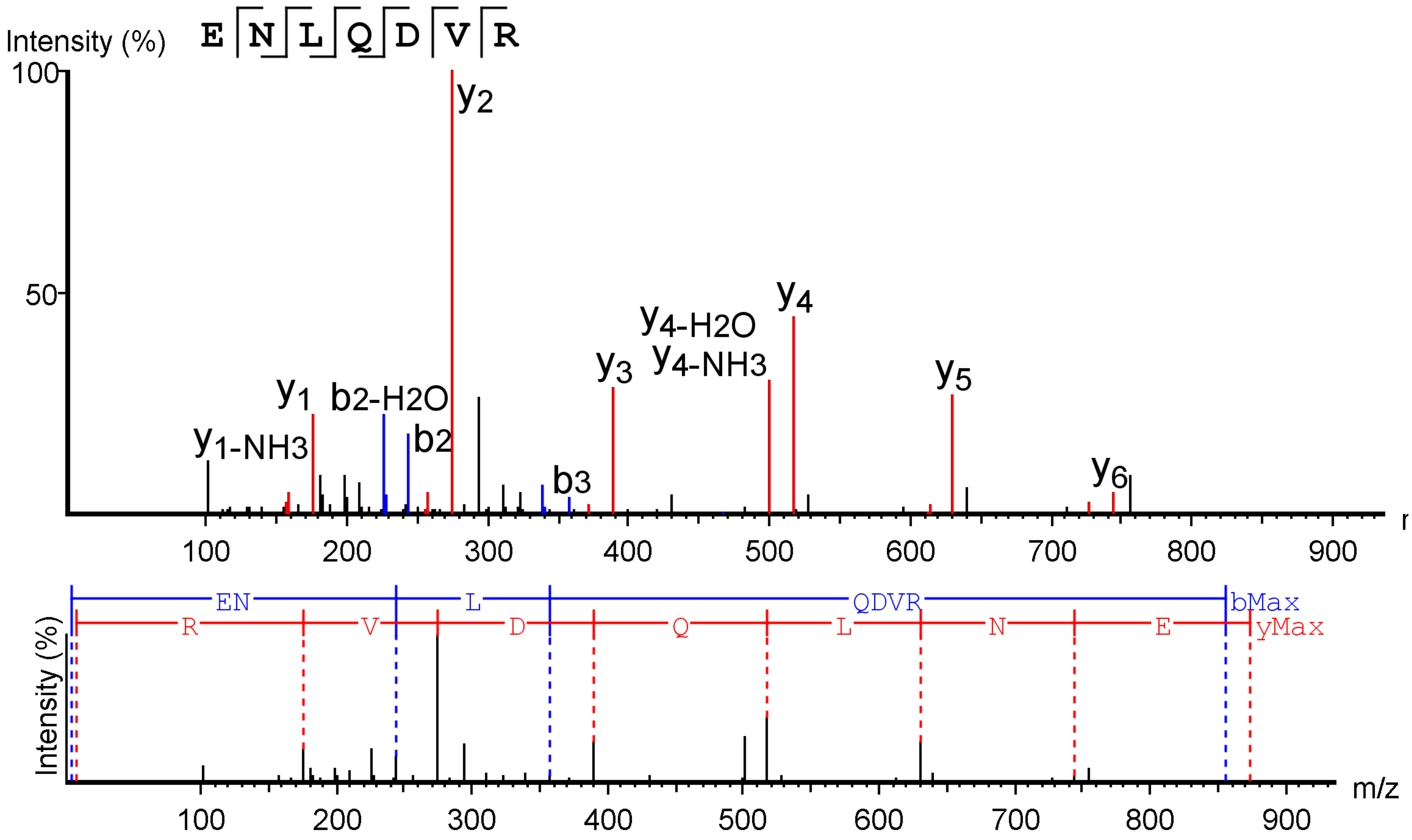

Figure 12. ***De novo*** **sequence analysis of the processed MS/MS spectra of** ***M. glareolus*** **tryptic peptide 873.4 m/z (Figure 2, t15)**

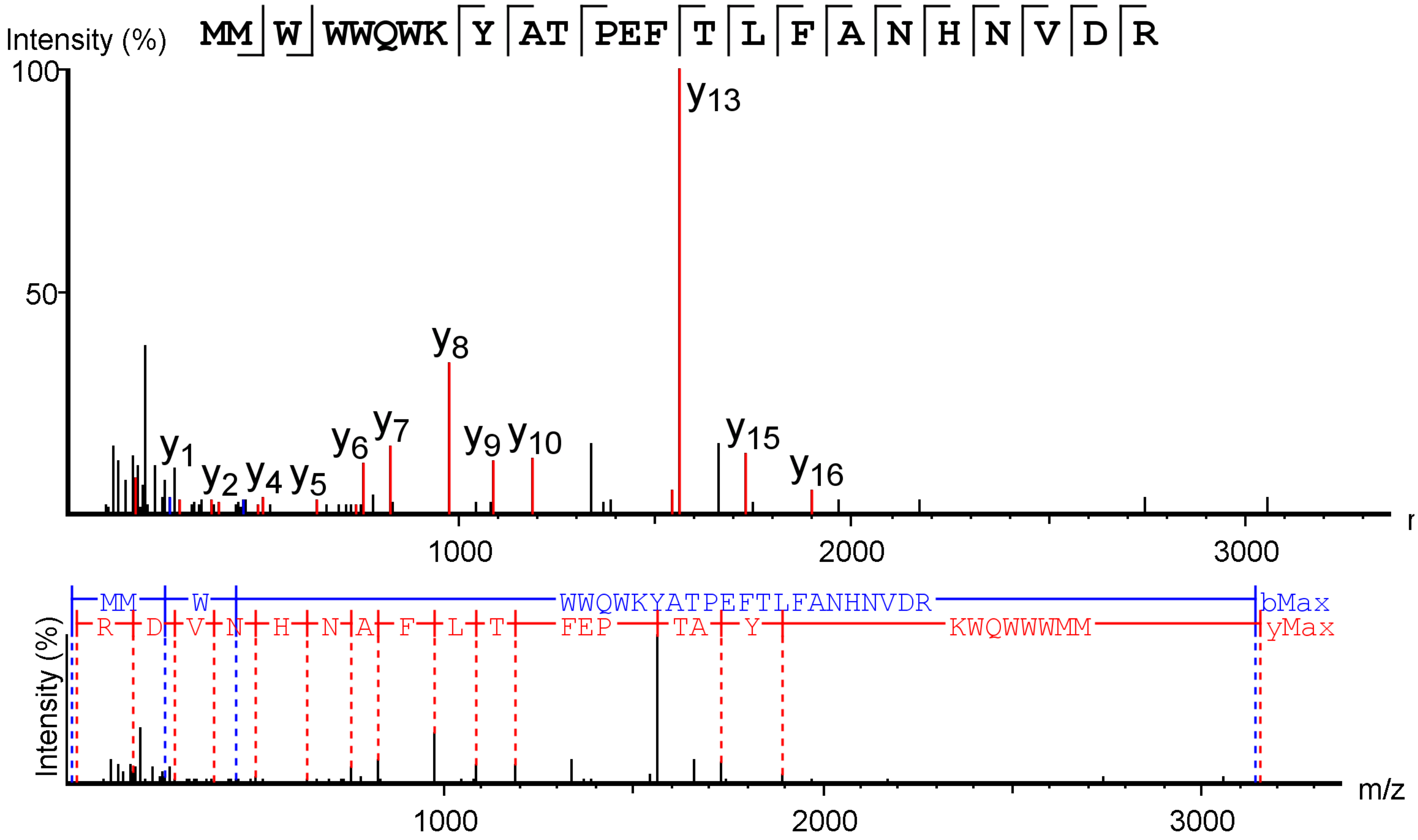

Figure 13. ***De novo*** **sequence analysis of the processed MS/MS spectra of** ***M. glareolus*** **tryptic peptide 3157.4 m/z (Figure 2, t9)**

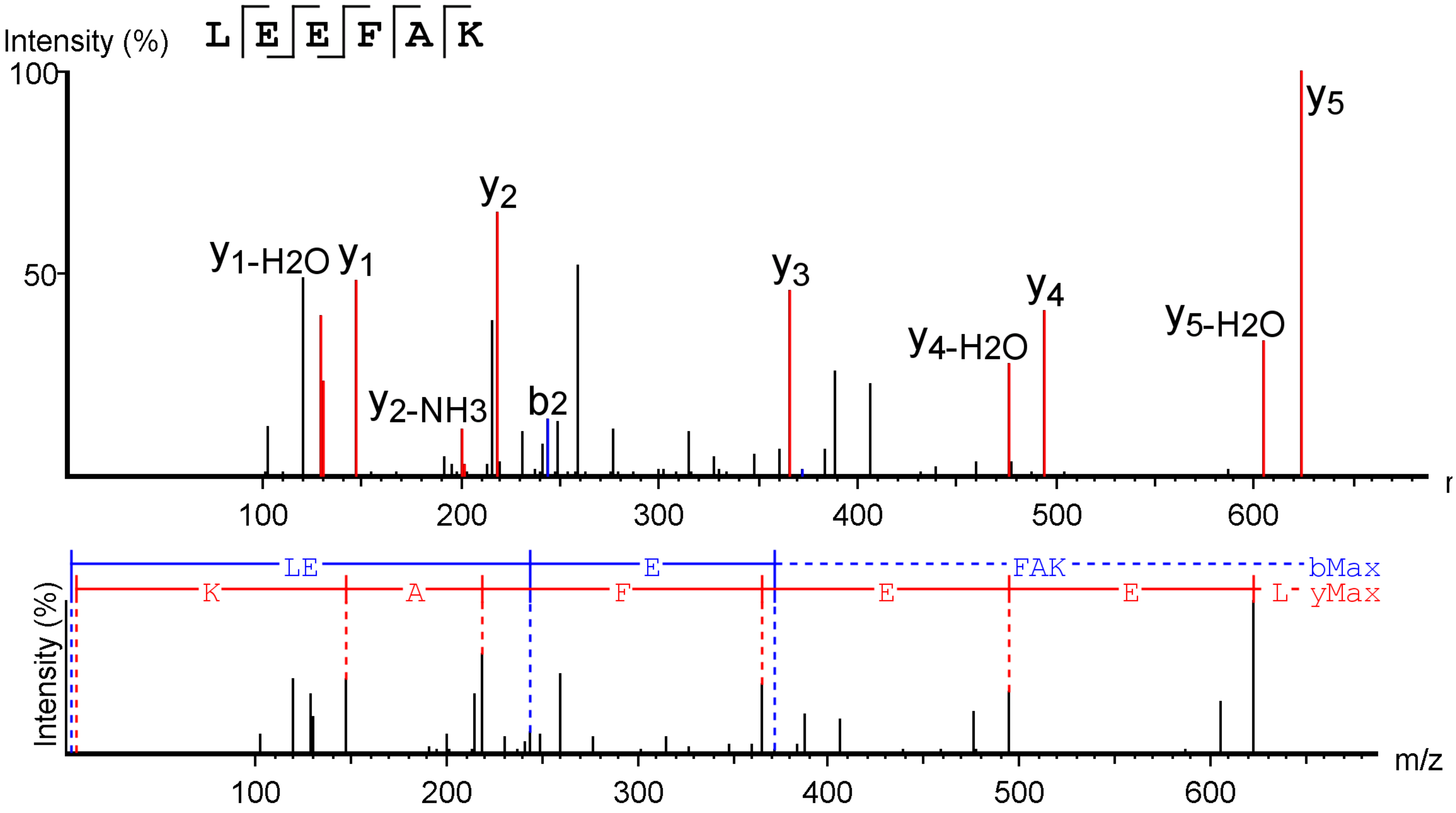

Figure 14. *De novo* sequence analysis of the processed MS/MS spectra of *M. glareolus* tryptic peptide 736.3 m/z (Figure 2, t13)

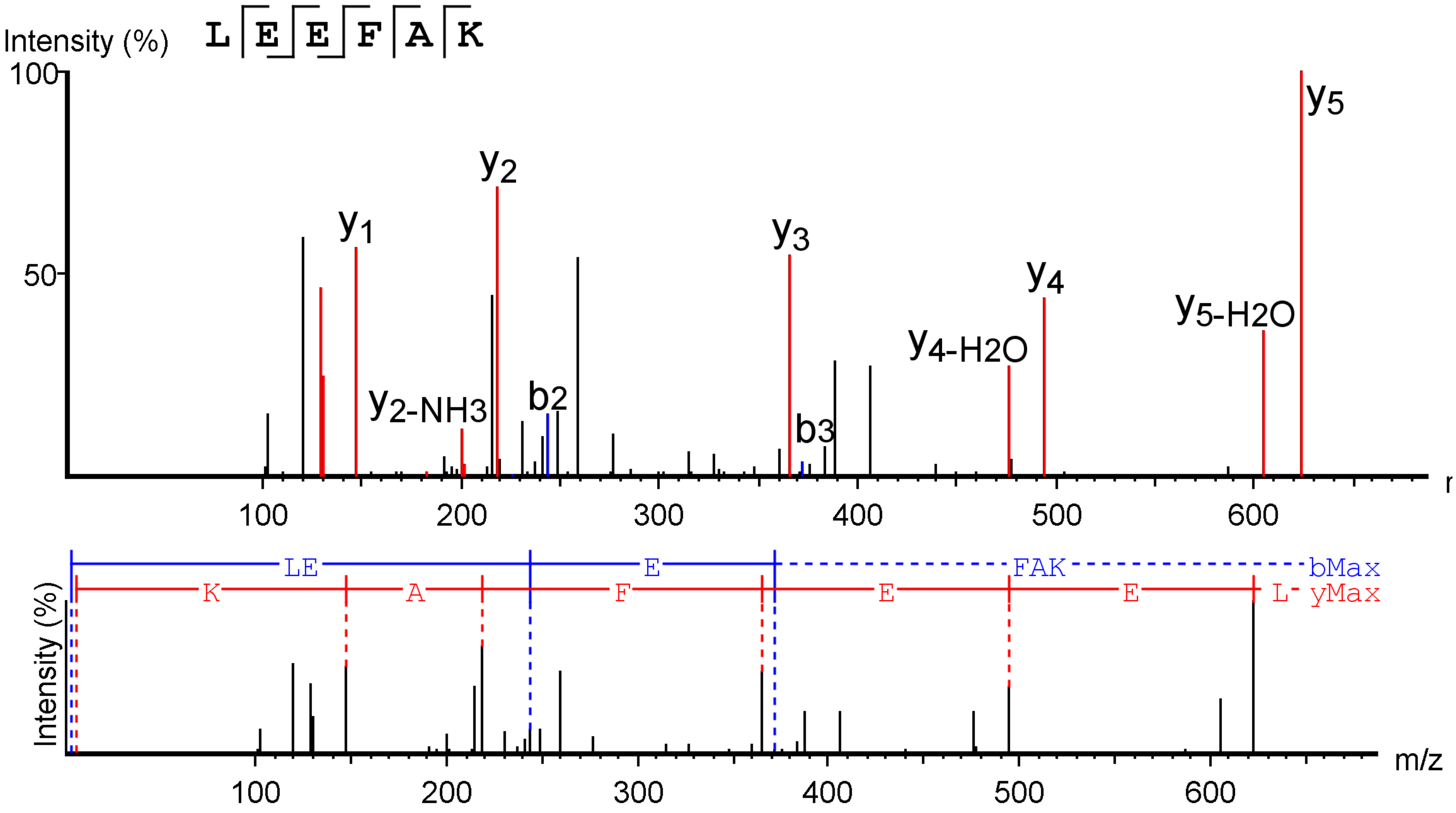

Figure 15. *De novo* sequence analysis of the processed MS/MS spectra of *M. glareolus* LysC peptide 736.3 m/z (Figure 2, t13)

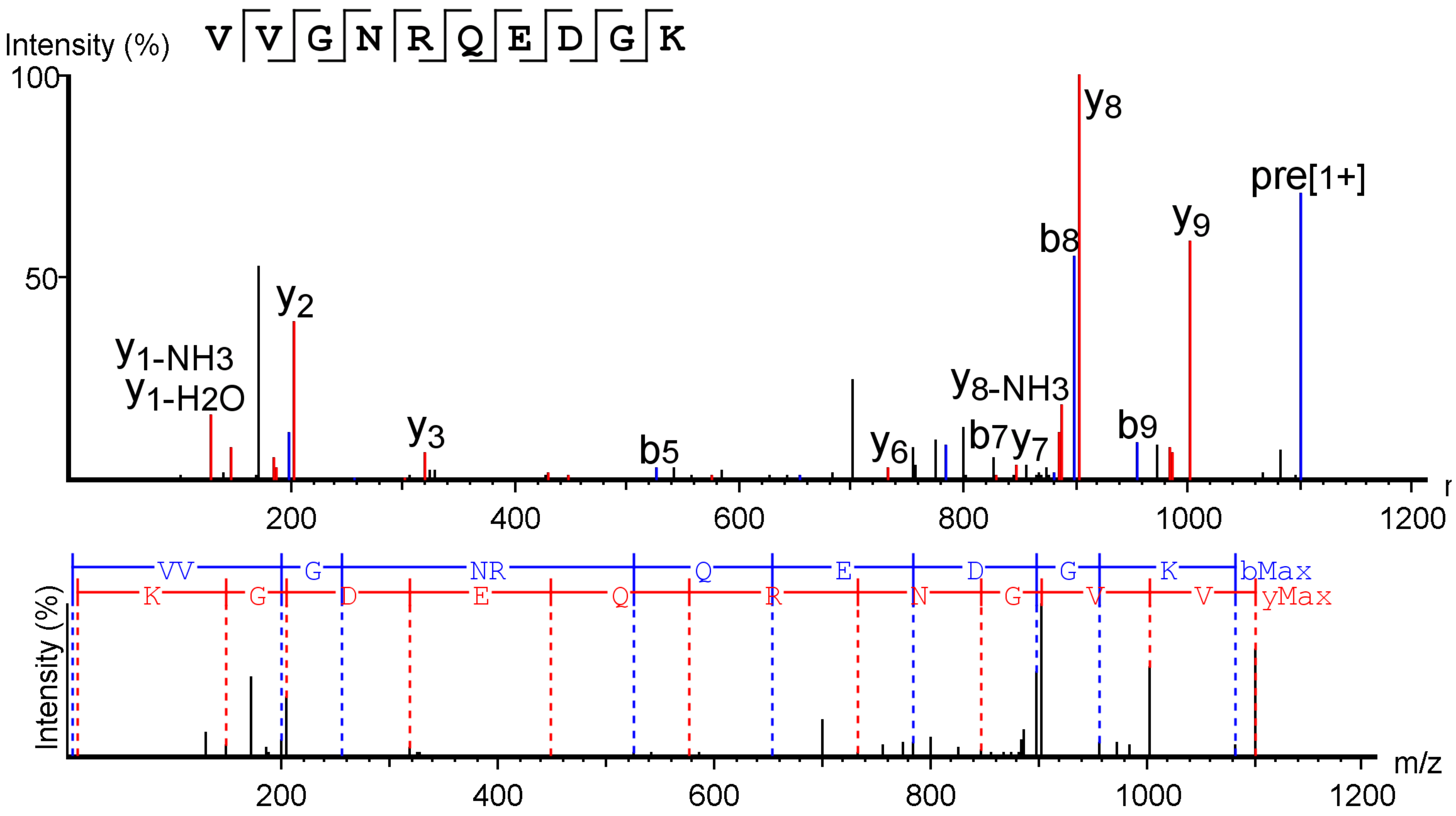

Figure 16. *De novo* sequence analysis of the processed MS/MS spectra of *M. glareolus* LysC peptide 1101.5m/z (Figure 2, c1)

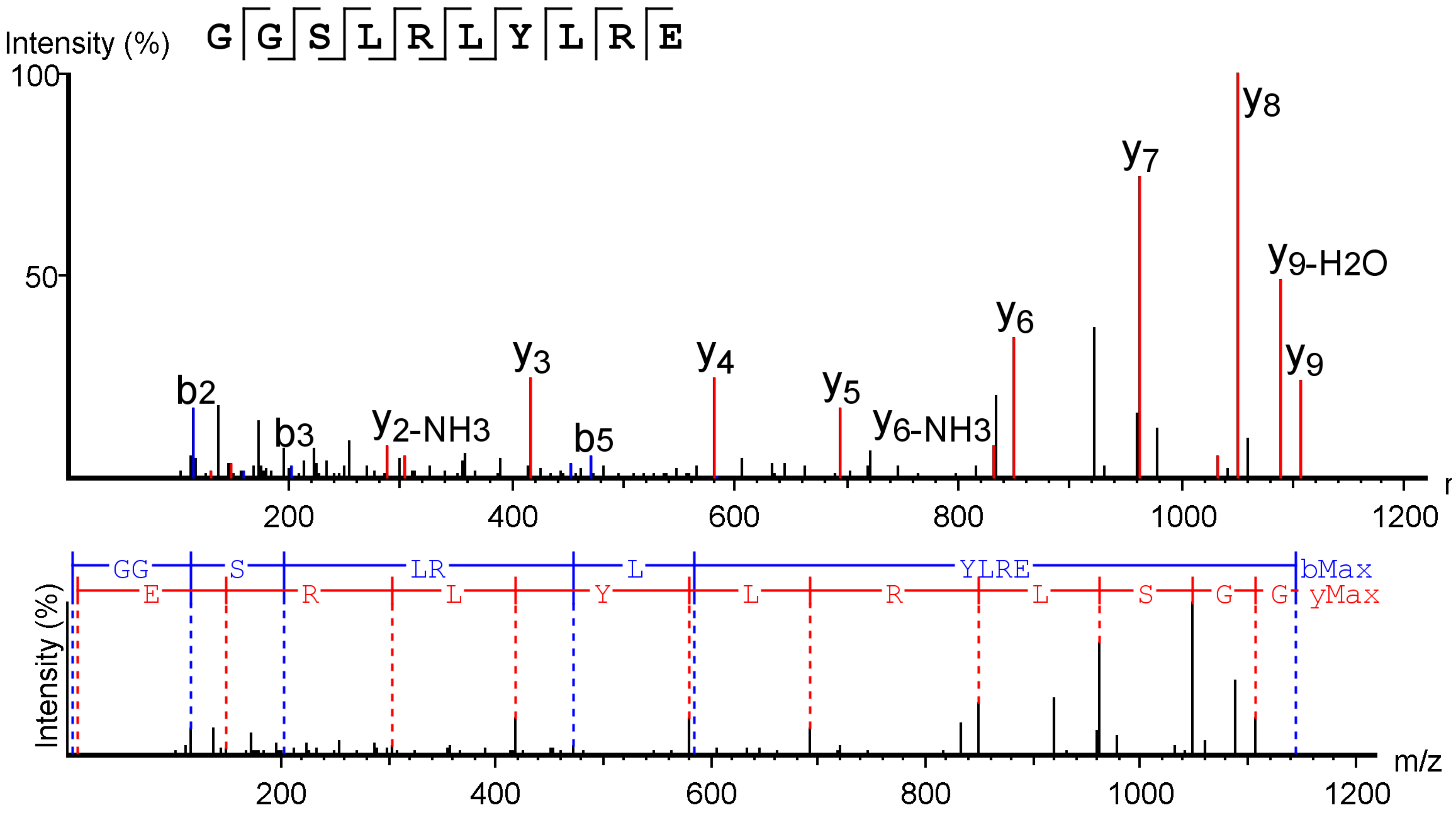

Figure 17. ***De novo*** **sequence analysis of the processed MS/MS spectra of** ***M. glareolus*** **GluC peptide 1163.6 m/z (Figure 2, g2)**

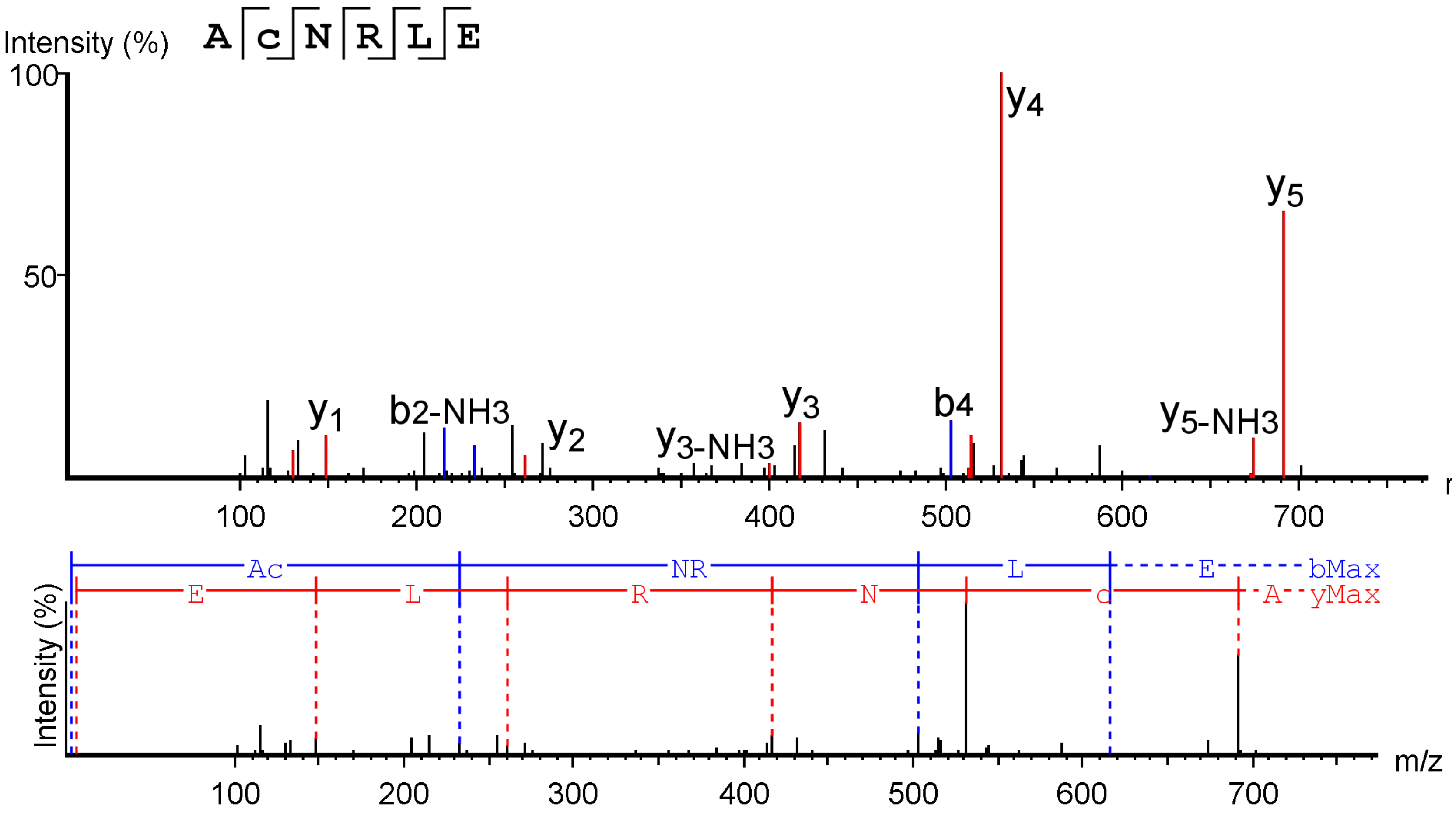

Figure 18. ***De novo*** **sequence analysis of the processed MS/MS spectra of** ***M. glareolus*** **GluC peptide 762.3 m/z (Figure 2, g1)**

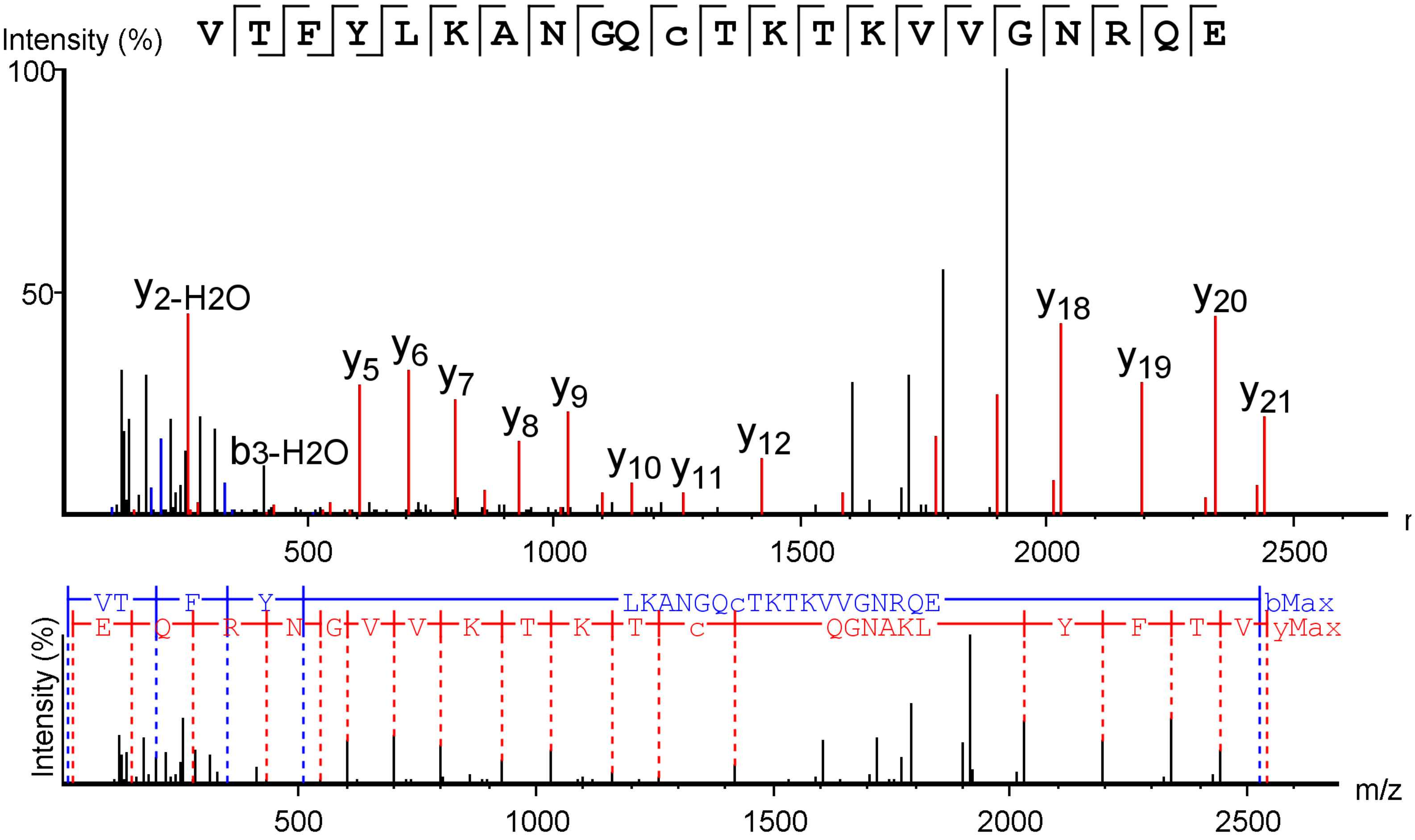

Figure 19. *De novo* **sequence analysis of the processed MS/MS spectra of** ***M. glareolus*** **GluC peptide 2541.3 m/z (Figure 2, g1)**

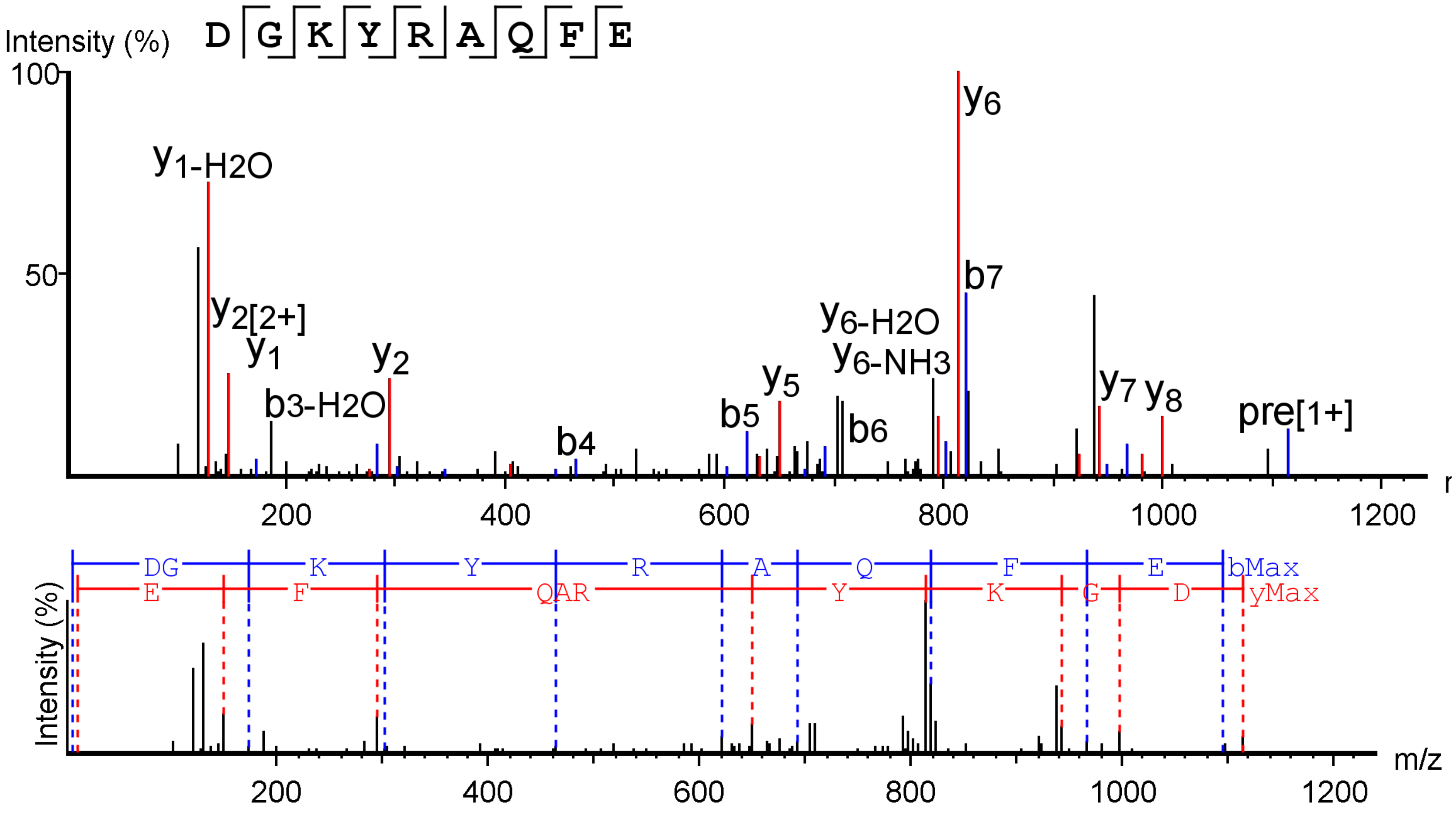

Figure 20. *De novo* **sequence analysis of the processed MS/MS spectra of** ***M. glareolus*** **GluC peptide 1113.5 m/z (Figure 2, g5)**

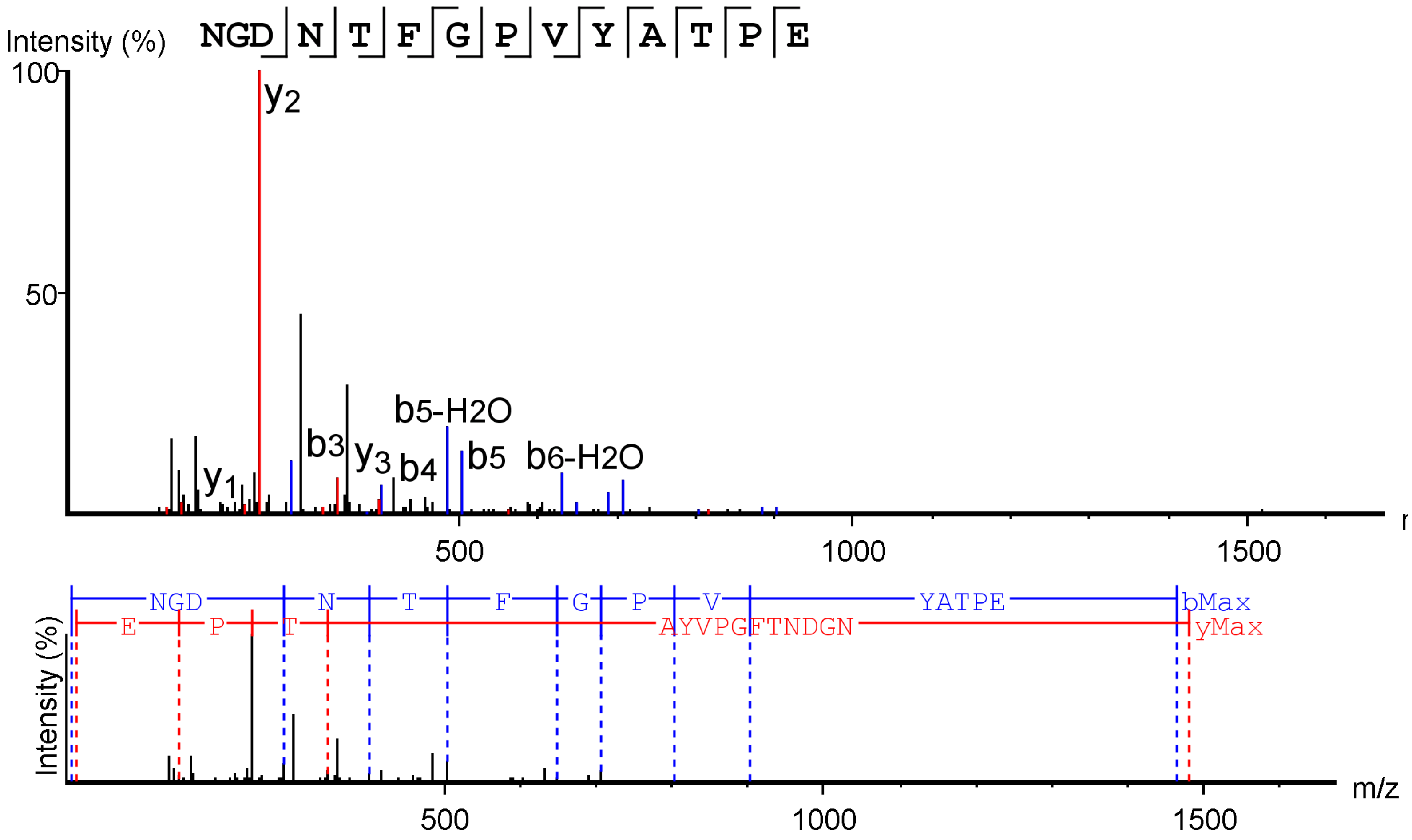

Figure 21. ***De novo*** **sequence analysis of the processed MS/MS spectra of *M. glareolus* GluC peptide 1481.6 m/z (Figure 2, g61)**

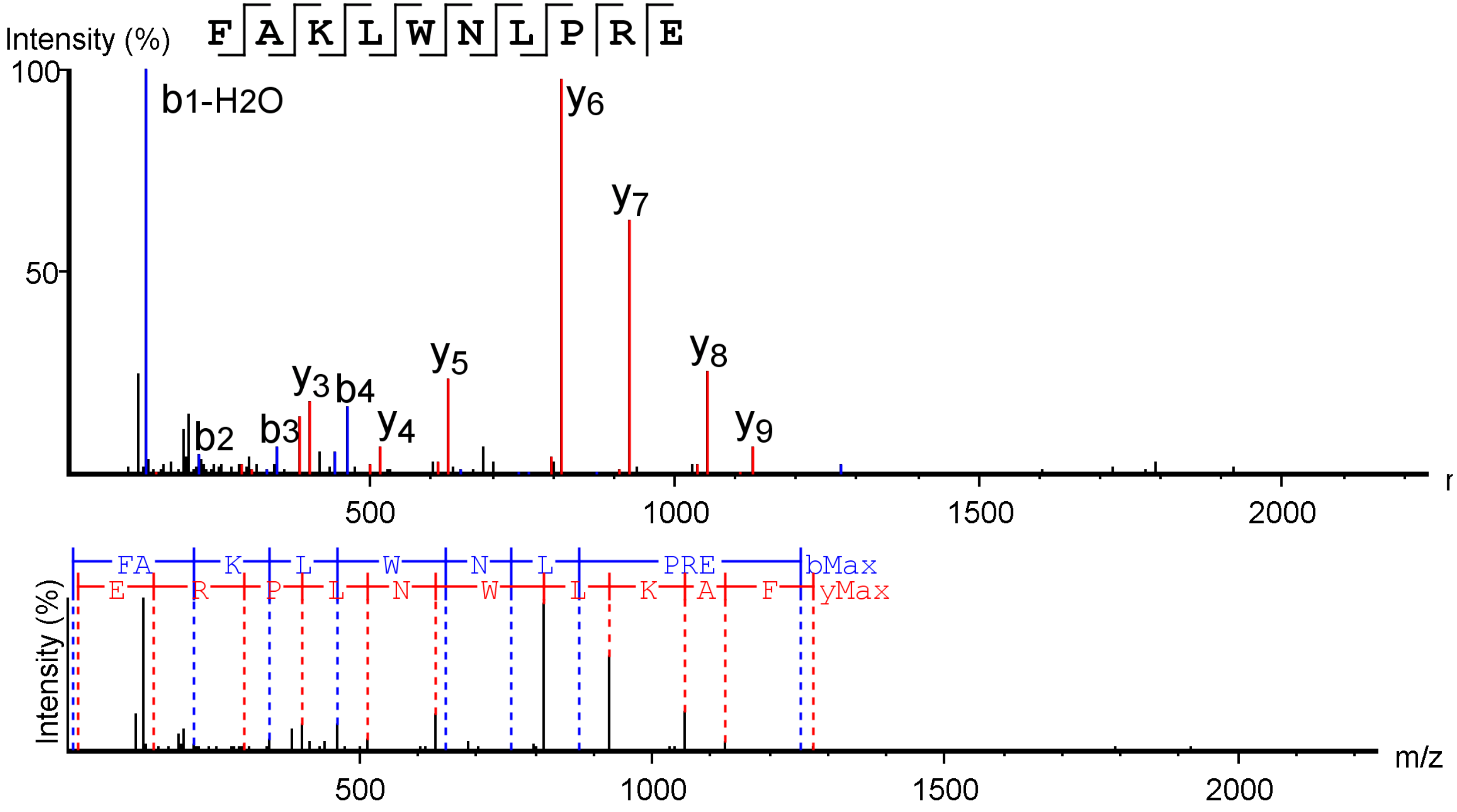

Figure 22. *De novo* **sequence analysis of the processed MS/MS spectra of** *M. glareolus* **GluC peptide 1273.7 m/z (Figure 2, g8)**

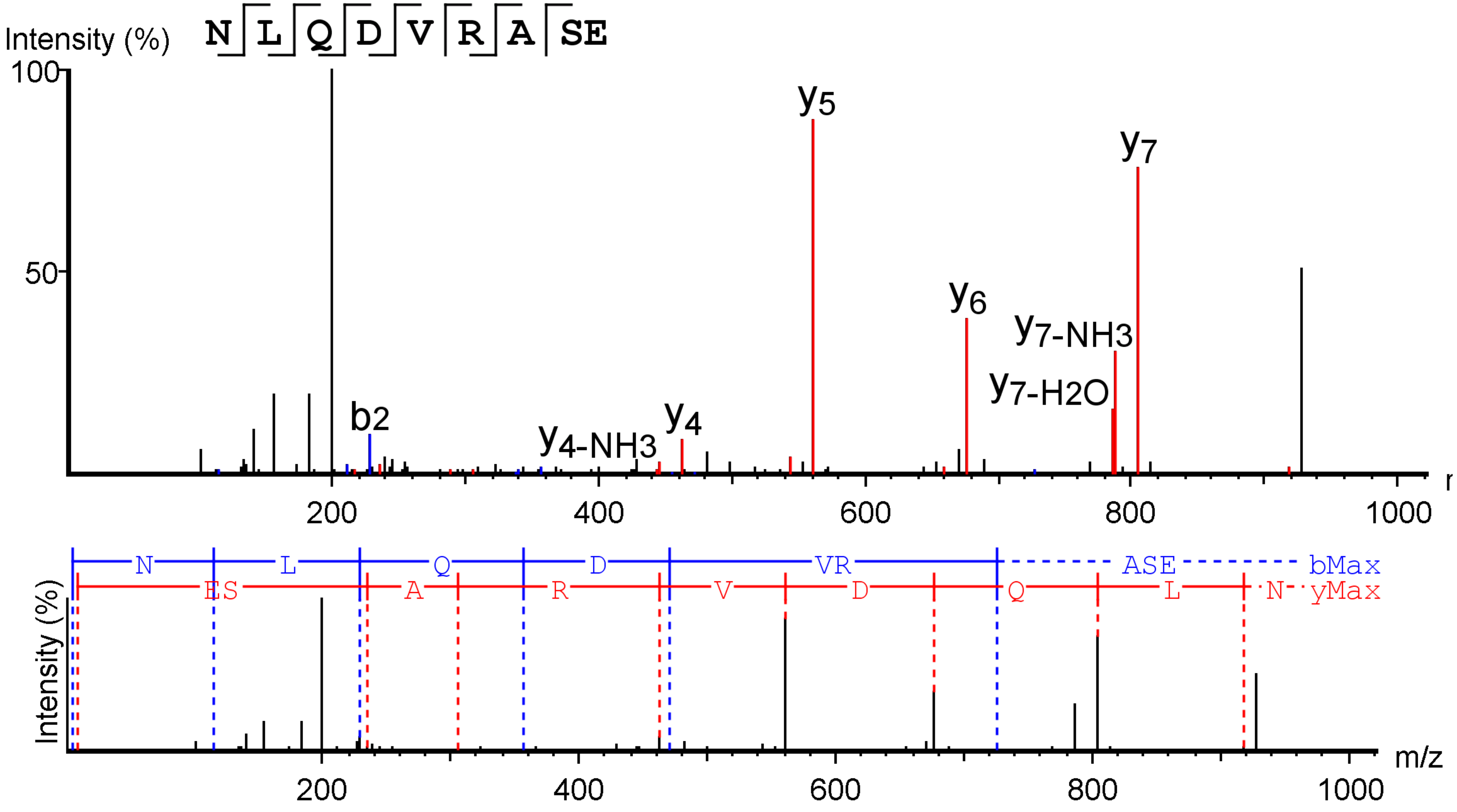

Figure 23. ***De novo*** **sequence analysis of the processed MS/MS spectra of** ***M. glareolus*** **GluC peptide 1031.5 m/z (Figure 2, g9)**

Supplementary material

Glareosin: a novel sexually dimorphic urinary lipocalin in the bank vole, *Myodes glareolus*

Grace M Loxley, Jennifer Unsworth, Michael J. Turton, Alexandra Jebb, Kathryn S Lilley, Deborah M Simpson, Daniel J. Rigden, Jane L. Hurst & Robert J. Beynon

S4. MS/MS spectra for resolution of ambiguous Leu/Ile peptide 'JYJRE'

The peptide marked with an asterisk in Figure 3 contains two Leu/Ile residues. The mass shift on metabolic labelling was consistent with a single residue being leucine, but the position could only be resolved by MS/MS analysis. The doublet y_1 and y_2 ions confirm the sequence as IYLRE.

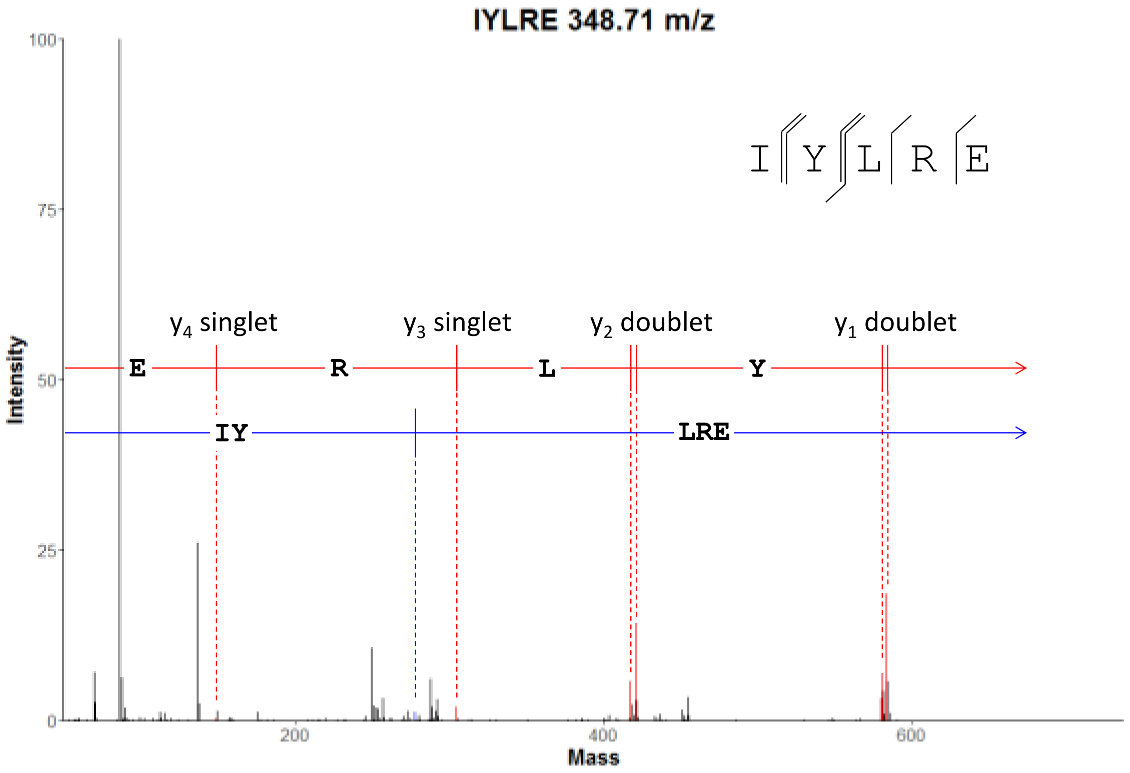

Supplementary material

Glareosin: a novel sexually dimorphic urinary lipocalin in the bank vole, *Myodes glareolus*

Grace M Loxley, Jennifer Unsworth, Michael J. Turton, Alexandra Jebb, Kathryn S Lilley, Deborah M Simpson, Daniel J. Rigden, Jane L. Hurst & Robert J. Beynon

S5. Alignment of glareosin with related lipocalins

Highlighted are the structural conserved GXW motif of lipocalins and the positions of the two disulphide bonds.

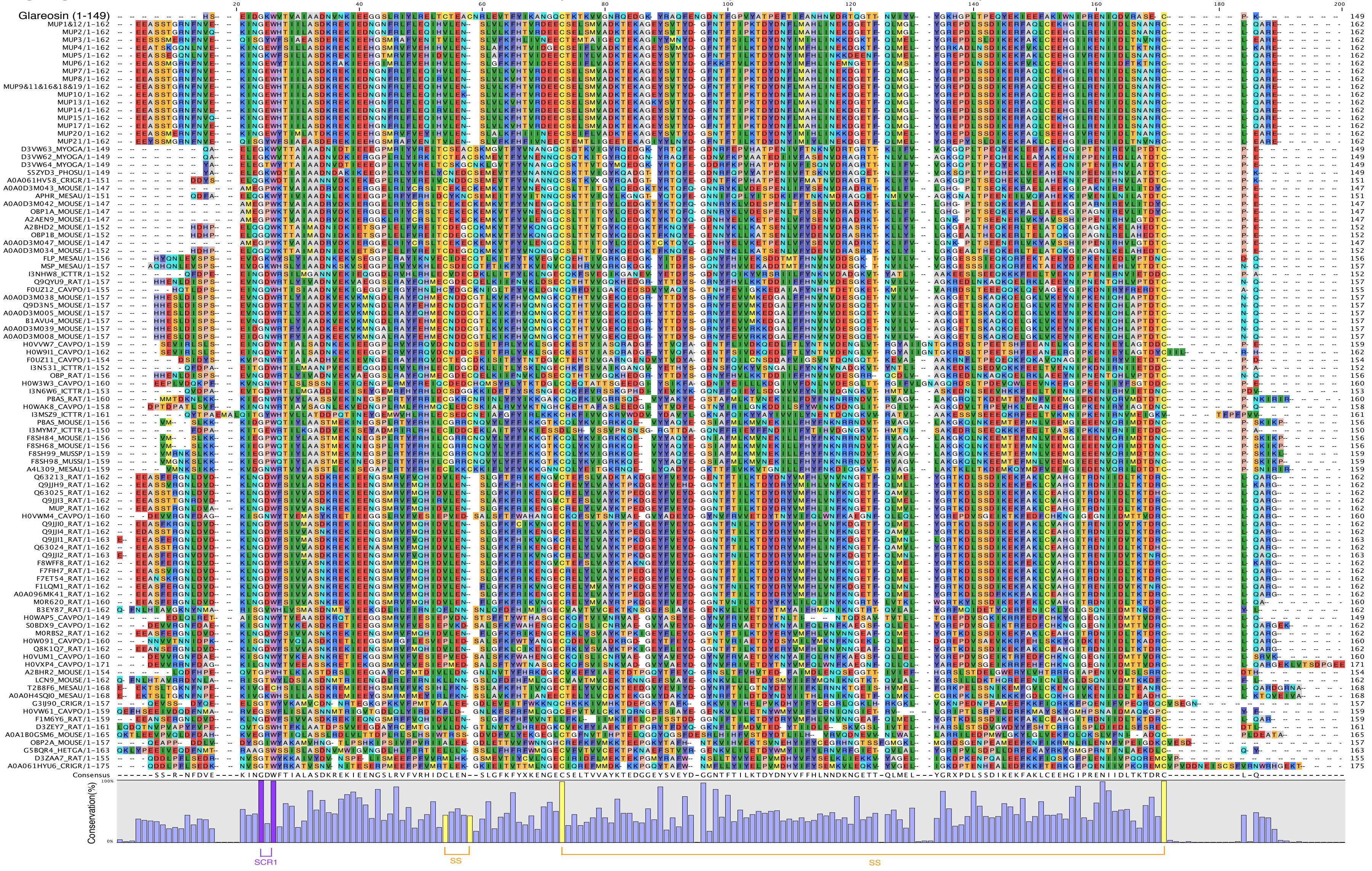

Supplement: Supplementary Methods S1; Supplementary Figure S2; Supplementary Figure S3; Supplementary Figure S4; Supplementary Figure S5 [file rsob170135supp1.pdf]
